# Aptamer-Modified Au Nanoparticles: Functional Nanozyme Bioreactors for Cascaded Catalysis and Catalysts for Chemodynamic Treatment of Cancer Cells

**DOI:** 10.1021/acsnano.2c05710

**Published:** 2022-10-26

**Authors:** Yu Ouyang, Michael Fadeev, Pu Zhang, Raanan Carmieli, Jiang Li, Yang Sung Sohn, Ola Karmi, Rachel Nechushtai, Eli Pikarsky, Chunhai Fan, Itamar Willner

**Affiliations:** † The Institute of Chemistry, The Hebrew University of Jerusalem, Jerusalem 91904, Israel; ‡ Department of Chemical Research Support, Weizmann Institute of Science, Rehovot 76100, Israel; ∇ School of Chemistry and Chemical Engineering, Frontiers Science Center for Transformative Molecules and National Center for Translational Medicine, Shanghai Jiao Tong University, Shanghai 200240, China; ∥ The Interdisciplinary Research Center, Shanghai Synchrotron Radiation Facility, Zhangjiang Laboratory, Shanghai Advanced Research Institute, Chinese Academy of Sciences, Shanghai 201210, China; ⊥ Institute of Life Science, The Hebrew University of Jerusalem, Jerusalem 91904, Israel; # The Lautenberg Center for Immunology and Cancer Research, IMRIC, The Hebrew University of Jerusalem, Jerusalem 91120, Israel

**Keywords:** Nanozyme, DNA nanotechnology, Peroxidase, Oxidase, Reactive oxygen species (ROS), Chemodynamic cancer therapy

## Abstract

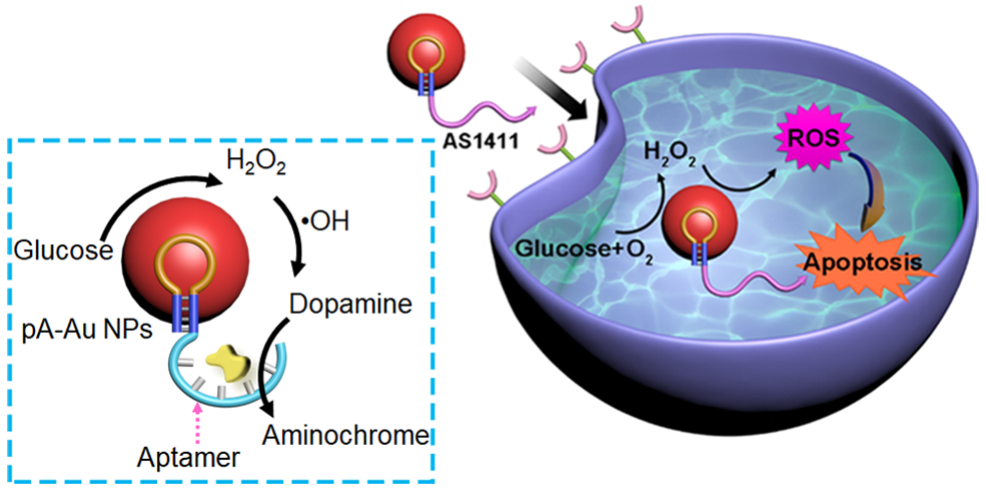

Polyadenine-stabilized Au nanoparticles (pA-AuNPs) reveal dual nanozyme catalytic activities toward the H_2_O_2_-mediated oxidation of dopamine to aminochrome and toward the aerobic oxidation of glucose to gluconic acid and H_2_O_2_. The conjugation of a dopamine-binding aptamer (DBA) to the pA-AuNPs yields aptananozyme structures catalyzing simultaneously the H_2_O_2_-mediated oxidation of dopamine to aminochrome through the aerobic oxidation of glucose. A set of aptananozymes consisting of DBA conjugated through the 5′- or 3′-end directly or spacer bridges to pA-AuNPs were synthesized. The set of aptananozymes revealed enhanced catalytic activities toward the H_2_O_2_-catalyzed oxidation of dopamine to dopachrome, as compared to the separated pA-AuNPs and DBA constituents, and structure–function relationships within the series of aptananozymes were demonstrated. The enhanced catalytic function of the aptananozymes was attributed to the concentration of the dopamine at the catalytic interfaces by means of aptamer–dopamine complexes. The dual catalytic activities of aptananozymes were further applied to design bioreactors catalyzing the effective aerobic oxidation of dopamine in the presence of glucose. Mechanistic studies demonstrated that the aptananozymes generate reactive oxygen species. Accordingly, the AS1411 aptamer, recognizing the nucleolin receptor associated with cancer cells, was conjugated to the pA-AuNPs, yielding a nanozyme for the chemodynamic treatment of cancer cells. The AS1411 aptamer targets the aptananozyme to the cancer cells and facilitates the selective permeation of the nanozyme into the cells. Selective cytotoxicity toward MDA-MB-231 breast cancer cells (*ca*. 70% cell death) as compared to MCF-10A epithelial cells (*ca*. 2% cell death) is demonstrated.

## Introduction

Substantial research efforts are directed to the development of inorganic, organic, or metal–organic framework nanoparticles (NMOFs) as catalysts mimicking native enzymes, *i.e.*, “nanozymes”.^1−3^ Inorganic catalysts, including metal oxides such as CeO_2_,^4^ V_2_O_5_,^5,6^ Fe_3_O_4_,^7−10^ and MoO_3_,^11^ metal nanoparticles such as Au,^12,13^ Ag,^14,15^ and Pt,^16,17^ carbon-based materials such as metal ions-modified carbon dots (C-dots)^18^ or graphene quantum dots,^19^ and composite nanoparticles such as Prussian Blue^20,21^ or Ag@Cu core–shell particles,^22^ reveal enzyme-like catalytic activities. Organic catalytic particles, including, for example, melamine^23^ or polydopamine^24^ nanoparticles, and NMOFs, such as Zr-based NMOFs modified with metal ions^25,26^ or MOF-818,^27^ have revealed nanozyme activities. Diverse enzyme activities have been emulated by nanozymes, including peroxidase,^28−30^ oxidase,^31−33^ laccase,^34^ superoxide dismutase,^35^ catalase,^4,36^ isomerase,^37^ and hydrolase^38,39^ activities. Also, hybrid multi-enzyme nanoparticle carriers or hybrids consisting of enzyme/nanozyme nanoparticles have been reported as composite nanoreactors for operating catalytic or biocatalytic cascades.^40,41^ Different applications of nanozymes^42,43^ or nanoreactor composites^44^ have been demonstrated, including their use as sensors^45,46^ and imaging agents,^47,48^ in medical applications such as cancer therapy,^42,49,50^ for treatment of diseases such as Alzheimer’s^51,52^ or Parkinson’s disease,^53,54^ and for catalytic release of cardiovascular drugs.^55^ In addition, nanozymes have been used as antibacterial and wound-healing agents by generating reactive oxygen species (ROS)^56^ and applied as catalysts for the degradation of pollutants.^57^

While nanozymes demonstrate enhanced stabilities as compared to native enzymes, they reveal lower catalytic activities and lack the stereoselectivity and chiroselectivity that are fundamental features of enzymes. As the high catalytic stereoselectivity and chiroselectivity functions of native enzymes originate from the well-defined structural and chiral architecture of the active site that provides cooperative binding of the substrate (high local concentration) and spatial proximity of the substrate to the catalytic site, efforts to mimic these features by nanozymes have been reported. These include the functionalization of the Cu^2+^-ion-modified C-dots nanozyme with β-cyclodextrin receptor binding units or the coating of nanocatalysts with molecularly imprinted matrices.^58^

The sequence-specific recognition properties of nucleic acids (aptamers)^59−61^ or catalytic properties of nucleic acids (DNAzymes)^62^ have been recently applied to develop nanozymes.^63,64^ The conjugation of aptamers to DNAzymes yielded enzyme-like conjugates (nucleoapzymes) revealing improved binding of the reaction substrate and enhanced catalytic and chiroselective properties.^65,66^ In addition, the functionalization of Cu^2+^-ion-modified C-dots with the dopamine aptamer or the tyrosinamide aptamer yielded highly active nanozymes for the oxidation of dopamine to aminochrome, the chiroselective oxidation of l-/d-DOPA to l-/d-dopachrome, and oxygen insertion into aryl C–H bonds of tyrosinamide for its oxidation into amidodopachrome. This class of aptamer-modified nanozymes was termed by us “aptananozymes”.^67^

Nucleic acid-modified gold nanoparticles (AuNPs) have found diverse applications,^68^ such as sensing,^69^ imaging,^70^ assembly of chiroplasmonic structures^71^ and switches,^72^ construction of optical nanodevices,^73^ and the use of their thermoplasmonic properties as nanothermometers.^74^ Recently, polyadenine-stabilized AuNPs (pA-AuNPs) demonstrated peroxidase-like nanozyme activities^75^ reflected by the oxidation of 2,2′-azino-bis(3-ethylbenzothiazoline-6-sulfonic acid) (ABTS^2–^) by H_2_O_2_ to form ABTS^•–^.

**Figure 1:**
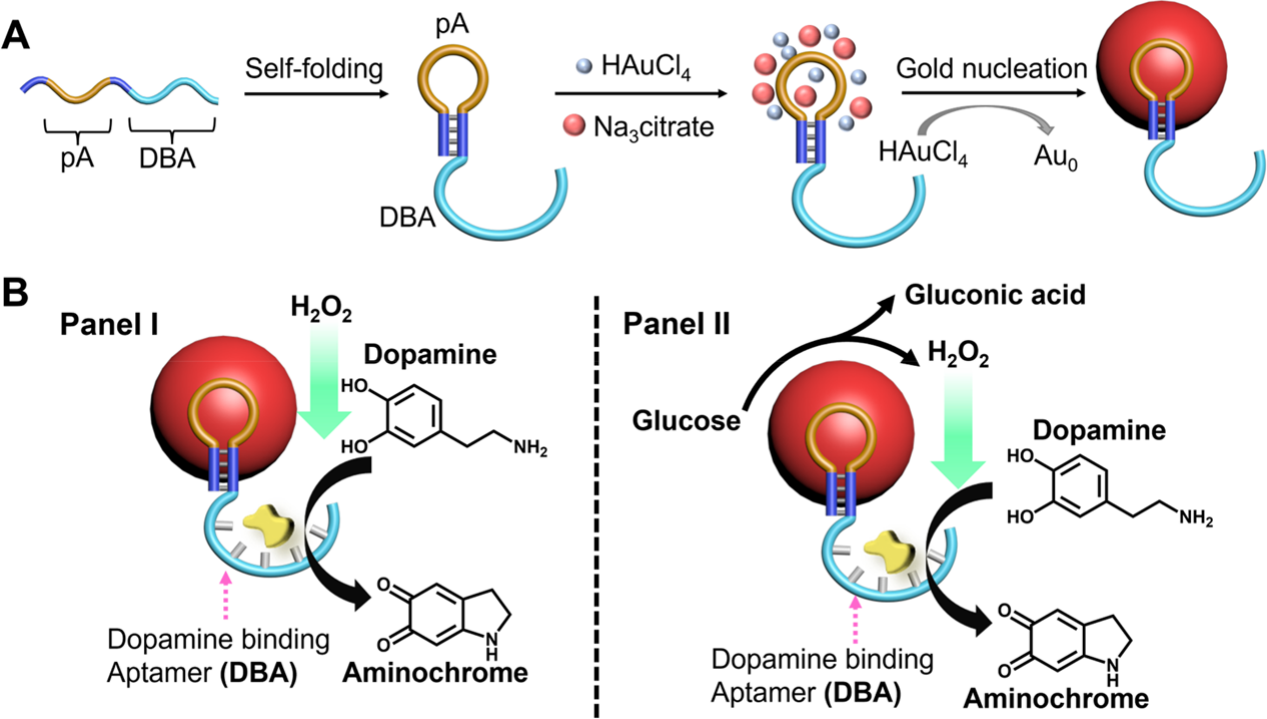
(A) Schematic synthesis of DBA-functionalized pA-AuNPs acting as aptananozymes. (B) The aptananozyme-catalyzed oxidation of dopamine to aminochrome by H_2_O_2_, Panel I and (Panel II) the aptananozyme-catalyzed oxidation of dopamine to aminochrome using the aptananozyme as a bioreactor, revealing dual consecutive catalytic functions consisting of the catalyzed aerobic oxidation of glucose to gluconic acid and H_2_O_2_, followed by the catalyzed oxidation of dopamine to aminochrome by the H_2_O_2_, Panel II.

Here we wish to report on the peroxidase-like nanozyme activity of pA-AuNPs toward the oxidation of dopamine to aminochrome by H_2_O_2_. Furthermore, we find that the pA-AuNPs act, also, as a nanozyme that catalyzes the aerobic oxidation of glucose to gluconic acid and H_2_O_2_. This allows the use of pA-AuNPs as a bioreactor nanozyme that catalyzes the cascaded aerobic oxidation of dopamine to aminochrome in the presence of glucose. That is, in contrast to native enzymes that reveal a dictated catalytic function, we find that a single AuNP nanozyme can catalyze two different catalytic transformations, *e.g.*, catalyzed oxidation of dopamine by H_2_O_2_ and aerobic oxidation of glucose. This not only demonstrates the diversity of nanozymes *vs.* native enzymes, but also allows us to apply a single nanozyme as a bioreactor that activates a cascaded biocatalytic process where the catalyzed aerobic oxidation of glucose to gluconic acid and H_2_O_2_ catalyzes oxidation of dopamine to aminochrome. The peroxidase activity of pA-AuNPs toward the oxidation of dopamine is, however, quite low. To enhance the nanozyme activity, we tether a series of dopamine-binding aptamers (DBAs) directly to their 3′- or 5′-end or through spacer units to yield aptananozyme bioreactors, Figure 1. The best aptananozyme reveals a 10-fold-enhanced oxidation of dopamine by H_2_O_2_ and a 13-fold-enhanced oxidation of dopamine in the presence of glucose, as compared to the separated nanozyme/aptamer units. In addition, we demonstrate the chiroselective oxidation of l-/d-DOPA to l-/d-dopachrome by H_2_O_2_ and the DBA-functionalized pA-AuNPs, aptananozyme, and the chiroselective oxidation of l-/d-DOPA to l-/d-dopachrome by glucose and the aptananozyme bioreactor. It should be noted that previous studies demonstrated the catalytic activities of “naked” AuNPs toward the aerobic oxidation of glucose,^31^ yet surface functionalization of the AuNPs led to catalytically inactive particles. Thus, the demonstration that pA-AuNPs retain their glucose oxidase nanozyme activities is significant and moreover important as it allows the conjugation of aptamer tethers to the pA-AuNPs that enable sequestered biocatalytic cascades by the aptananozyme reactor and aptamer-guided therapeutic applications (*vide infra*) by the aptananozyme. The mechanism of the DBA-functionalized pA-AuNPs-catalyzed oxidation of dopamine by H_2_O_2_ involves the generation of ^•^OH as ROS intermediates.

The glucose-mediated generation of ^•^OH by the pA-AuNPs is used to engineer AS1411 aptamer-functionalized pA-AuNPs as functional aptananozymes for the chemodynamic treatment of MDA-MB-231 cancer cells. The cytotoxicity of the AS1411/pA-AuNPs toward MDA-MB-231 cancer cells and toward MDA-MB-231 xenogragt tumor-bearing mice is addressed.

## Results and Discussion

The pA-AuNPs were synthesized according to the reported procedure,^75^Figure 1A. (For details and characterization, see the Supporting Information, Figures S1–S3.) The resulting pA-AuNPs reveal peroxidase-like activities and result in the oxidation of dopamine by H_2_O_2_ to form aminochrome, Figure S4. In addition, we find that pA-AuNPs reveal glucose oxidase activities and, in the presence of glucose, catalyze the aerobic oxidation of glucose to gluconic acid and H_2_O_2_, Figure S5. The dual catalytic activities of the pA-AuNPs allowed, then, the use of the pA-AuNPs as a nanozyme bioreactor for the catalyzed aerobic oxidation of dopamine to aminochrome in the presence of glucose, Figure S6. In this system, the aerobic oxidation of glucose to gluconic acid and H_2_O_2_, and the resulting H_2_O_2_ in the vicinity of the catalytic particles, allows the subsequent cascade oxidation of dopamine to aminochrome. (For mechanistic characterization of the nanozyme bioreactor, *vide infra*.)

While the pA-AuNPs reveal dual catalytic activities and establish a nanozyme bioreactor function, the effectiveness of the system toward the H_2_O_2_-catalyzed oxidation of dopamine to aminochrome and the cascaded aerobic oxidation of dopamine, in the presence of glucose, is moderate, as compared to other peroxidase-mimicking nanozymes. The importance of the systems rests on the dual catalytic bioreactor activities of the particles. To improve the catalytic activities of the pA-AuNPs system, we applied the concept of aptananozyme, where the DBA was tethered to the pA-AuNPs. In these systems, the DBA tethered to the particles concentrate the dopamine substrate at the aptamer-binding site, thereby allowing the effective utilization of H_2_O_2_, Figure 1B, Panel I and Panel II.

**Figure 2:**
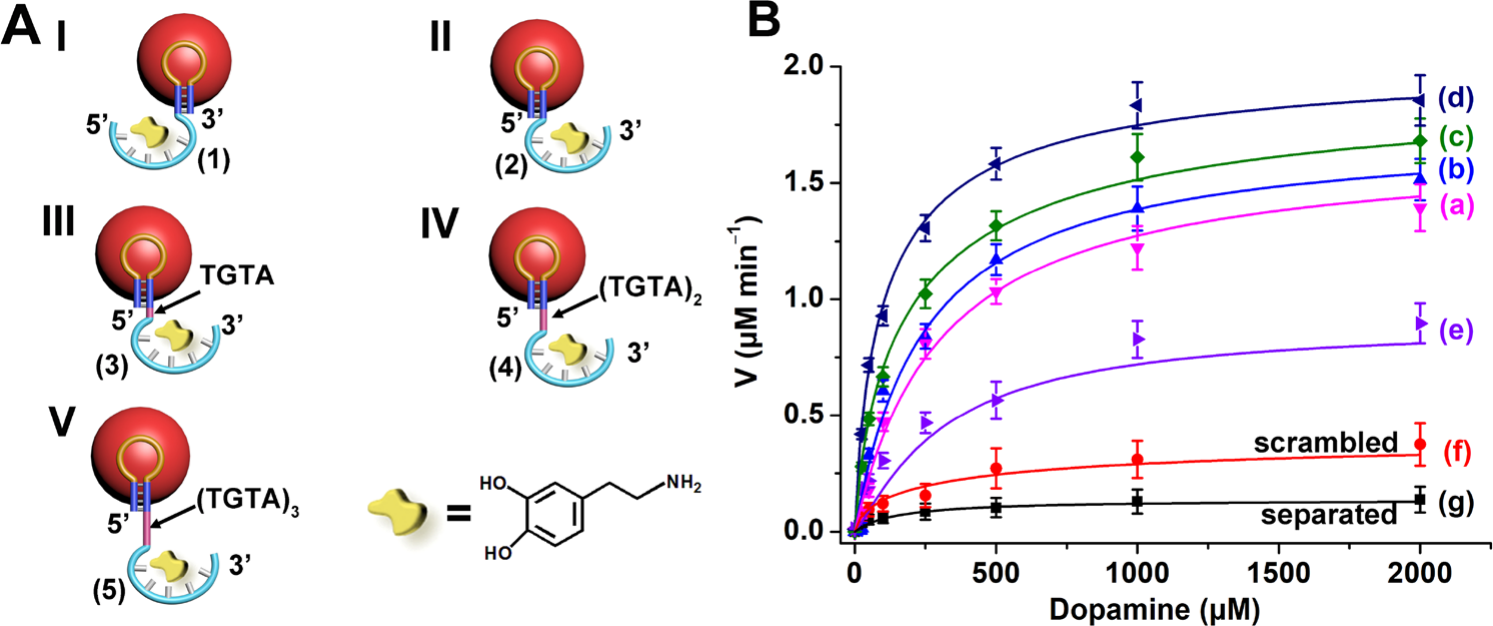
(A) Schematic structures of the set of DBA-functionalized pA-AuNPs, i.e., aptananozymes, acting as catalysts for the oxidation of dopamine to aminochrome by H_2_O_2_. (B) Rates of dopamine oxidation to aminochrome in the presence of H_2_O_2_ by (a–e) aptananozymes I–V, (f) the pA-AuNPs functionalized with scrambled DBA (**2a**), and (g) the pA-AuNPs separated with DBA (**2**). All systems are composed of a MES buffer solution, pH = 5.5, 5 mM MgCl_2_, 100 mM NaCl, that includes the 5 nM aptananozyme and 5 mM H_2_O_2_. Error bars derived from *N* = 3 experiments.

A series of DBAs were conjugated to the pA units stabilizing the AuNPs, Figure 2A. These included the conjugation of the 3′-end DBA (**1**) to the pA stabilizer units (aptananozyme I) and the 5′-end aptamer sequence (**2**) to the pA sequence (aptananozyme II), and the 5′-end aptamer tethered to the pA sequence *via* a four-base spacer TGTA bridge (**3**), an eight-base spacer (TGTA)_2_ (**4**), and a 12-base spacer (TGTA)_3_ sequence (**5**) (aptananozymes III–V, respectively). (The aptamer and aptamer–spacer sequences elongated at the respective 3′- or 5′-end of the pA hairpin were used to synthesize the pA-AuNPs.) All pA-AuNPs include an identical loading of the modified pA-aptamer strands on the AuNPs (∼1:1 molar ratio between the AuNPs and the pA nucleic acid stabilizer, Figure S1). Figure 2B shows the oxidation rates of dopamine to aminochrome in the presence of variable concentrations of dopamine (and excess of 5 mM H_2_O_2_) using the aptananozyme I, curve (a), aptananozyme II, curve (b), aptananozyme III, curve (c), and aptananozyme IV and V, curves (d) and (e), respectively. The time-dependent absorbance changes corresponding to aminochrome formation were applied to follow the kinetics of dopamine oxidation by the respective aptananozymes. For comparison, the rate of oxidation of dopamine by H_2_O_2_ in the presence of the pA-AuNPs modified with the nucleic acid (**2a**), consisting of the scrambled bases of DBA, is presented in curve (f), and the rate of oxidation of the dopamine by the separated pA-AuNPs/aptamer constituents in the presence of H_2_O_2_ is displayed in curve (g). The catalytic rates of the aptamer-modified nanozymes reveal a Michaelis–Menten-type kinetic behavior and reach saturation levels, consistent with the saturation of the aptamer-binding receptor sites. All aptamer-modified nanozymes show substantially enhanced catalytic oxidation of dopamine, as compared to the control systems of the separated nanozyme/aptamer couple or the nanozyme conjugated by scrambled bases aptamer (**2a**). The enhancement of the dopamine oxidation rate by the aptamer-modified nanozymes follows the order I < II < III < IV, indicating the following: (i) The tethering of the 5′-end to the pA-AuNPs yields a nanozyme of slightly higher catalytic activity as compared to the 3′-end-modified aptamer tethered to the pA-AuNPs. (ii) The introduction of a spacer unit bridging the 5′-end of the aptamer to the pA-AuNPs improves the catalytic performance of the nanozyme, and the eight-bases-bridged spacer leads to superior catalytic properties of the nanozyme IV > III. Nonetheless, further elongation of the spacer bridge to 12 bases decreases the catalytic performance of the nanozyme V < IV. The catalytic properties of the aptamer-functionalized pA-AuNPs nanozymes follow the binding properties between the aptamer–pA-AuNPs conjugates and dopamine substrate. (iii) The kinetic features, *K*_M_ and *V*_max_, associated with the different aptamers-functionalized pA-AuNPs are summarized in Table 1. The dissociation constants (*K*_d_) of different aptananozymes to dopamine substrates are also included in Figure S7 and Table 1. The results introduce important functions of the aptamer-modified AuNPs catalysts (aptananozymes) that can be summarized as follows: (i) The different aptananozymes reveal enhanced catalytic activities toward the oxidation of dopamine, as compared to the separated aptamer and pA-modified AuNPs. The catalytic activities of the aptananozyme follow the order I < II < III < IV. The superior **(4)**-modified pA-AuNPs reveal a 10-fold enhanced activity as compared to the separated aptamer/pA-AuNPs constituents. The enhanced catalytic activities of the aptananozymes are attributed to the binding of dopamine to the aptamer units resulting in the local concentration of the reaction substrate (molarity effect) at the catalytic interface. That is, the affinity binding of dopamine to the aptamer receptor controls the catalytic efficacies of the aptananozymes, similar to the functions of native enzymes, where the affinity binding of the substrates guides the biocatalytic performance. (ii) The order of the catalytic activities follows the order of disassociation constants to the aptamer units. The introduction of the oligonucleotide-TGTA units improves the binding features of the aptamer toward dopamine. Presumably, the AuNPs surface perturbs the association of dopamine to the aptamer-binding sites, and the spacer units added to the aptamer scaffolds introduce flexibility into the aptamer sequence that eliminates the steric binding perturbation toward the ligand introduced by the particles. (iii) For the long 12 (TGTA)_3_ bridge, a decrease in the catalytic activity is observed. This is attributed to the spatial separation of the substrate from the catalytic interface. Similar steric effects of long spacer bridges were observed for other nucleoapzyme^66^ and aptananozyme systems.^67^ (iv) The random sequence aptamer-modified pA-AuNPs, curve (v), reveal slightly enhanced oxidation of dopamine, as compared to the separated pA-AuNPs/aptamer constituents. This slight rate enhancement is attributed to the electrostatic concentration of the positively charged dopamine at the catalytic interface. (For further mechanistic studies addressing the participation of hydroxyl radicals ^•^OH in the pA-AuNPs-catalyzed oxidation of dopamine to aminochrome, *vide infra*.)

**Table 1:** Kinetic Parameters Associated with the Set of Aptananozymes I–V and Control Systems, and Dissociation Constants of the Respective Dopamine and Aptananozymes^a^

aptananozyme	*V*_max_ (μM min^–1^)	*K*_M_ (μM)	*k*_cat_ (s^–1^)	*K*_d_ (μM)
IV	2.05 ± 0.11	113 ± 13	3.8 ± 1.0	0.76 ± 0.03
III	1.95 ± 0.22	207 ± 67	6.5 ± 0.8	0.95 ± 0.04
II	1.73 ± 0.26	247 ± 36	5.7 ± 0.8	1.20 ± 0.06
I	1.66 ± 0.13	305 ± 67	5.5 ± 0.9	1.65 ± 0.03
V	0.89 ± 0.31	310 ± 56	3.0 ± 0.5	0.93 ± 0.05
scrambled DBA/pA-AuNPs	0.43 ± 0.05	334 ± 89	1.4 ± 0.6	–
separated DBA/pA-AuNPs^b^	0.2 ± 0.06	–	0.7 ± 0.08	–

a All experiments were performed in a 5 mM MES buffer solution, pH 5.5, that included 5 mM MgCl_2_, 100 mM NaCl, and 5 nM of the respective aptananozymes or control system and 5 mM H_2_O_2_. Error bars derived from *N* = 3 experiments.

b The separated pA-AuNPs/DBA system shows pseudo-first-order kinetics: *k* = 0.7 s^–1^.

**Figure 3:**
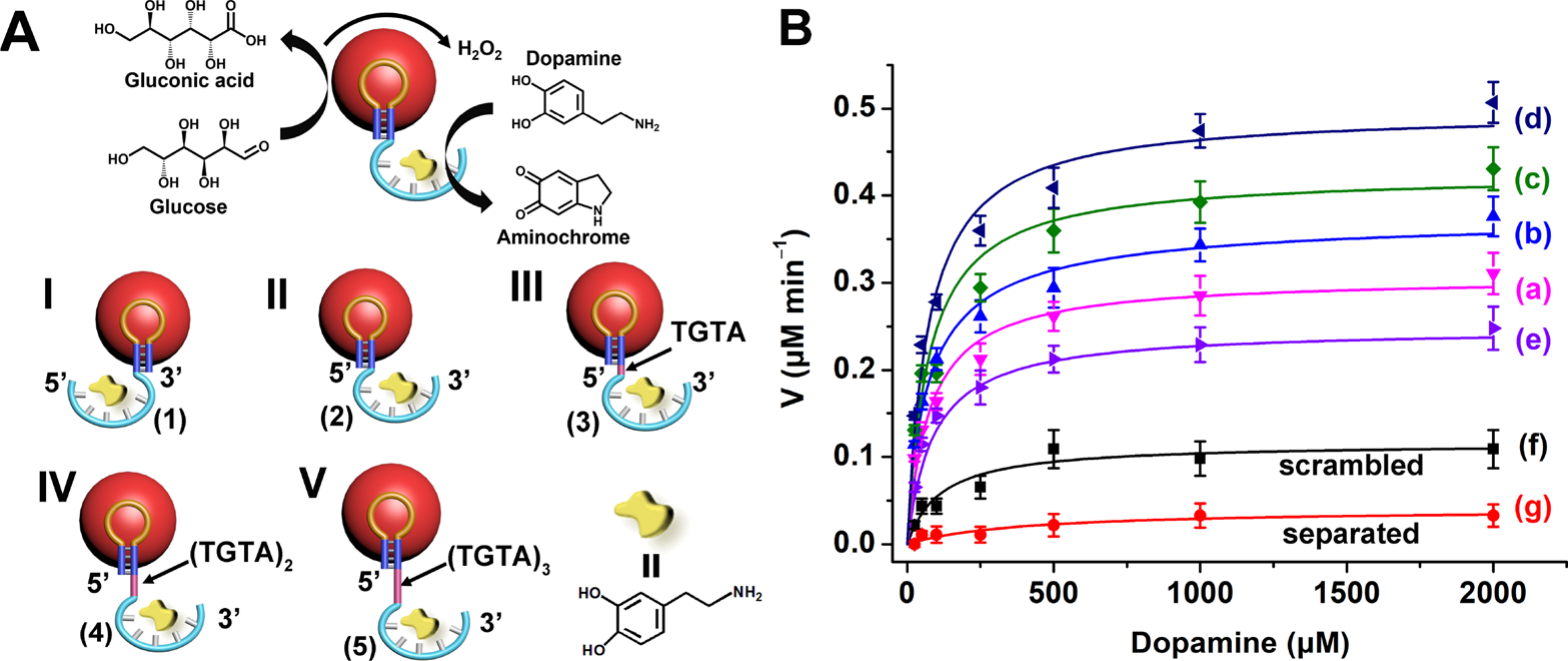
(A) Schematic presentation of the cascaded oxidation of dopamine to aminochrome by the aptamer-modified pA-AuNPs, i.e., the aptananozyme bioreactor system, using the catalyzed aerobic oxidation of glucose as the source of the intermediate H_2_O_2_ oxidant. The set of aptananozymes I–V was used to catalyze the cascaded process. (B) Rates of dopamine oxidation to aminochrome in the presence of glucose by (a–e) the aptananozyme in configurations I–V, (f) the pA-AuNPs functionalized with scrambled DBA (**2a**), and (g) the pA-AuNPs separated with DBA (**2**). All systems consist of a 5 mM MES buffer solution, pH = 5.5, 5 mM MgCl_2_, 100 mM NaCl, that includes the 5 nM aptananozyme and 50 mM glucose. Error bars derived from *N* = 3 experiments.

The DBA-functionalized pA-AuNPs, aptananozymes, reveal oxidase-like catalytic activities toward the oxidation of glucose, Figure S8, and the accompanying discussion depicts the glucose oxidase activities of the aptananozyme IV. In this experiment, the aerobic oxidation of glucose yields gluconic acid and H_2_O_2_, and the resulting H_2_O_2_ is quantitatively probed by the H_2_O_2_ oxidation of Amplex Red to the fluorescent Resorufin. All DBA-functionalized pA-AuNPs, i.e., aptananozymes I–V, reveal similar glucose oxidase activities, and their activities are very similar to the oxidase activity of the pA-AuNPs lacking the conjugated DBA aptamer. That is, the glucose oxidase activities of the pA-AuNPs are not influenced by the coupled DBA aptamer sequences, Figure S9. Realizing that the series of DBA-conjugated pA-AuNPs, aptananozymes I–V, exhibit dual nanozyme catalytic functions toward the aerobic oxidation of glucose and peroxidase activities toward the oxidation of dopamine by H_2_O_2_ to aminochrome, the set of aptananozymes was applied as bioreactor systems to guide the cascaded catalytic oxidation of dopamine by the aerobic oxidation of glucose, Figure 3A.

Figure 3B and Table 2 show the rates of dopamine oxidation using the different aptananozyme bioreactors I–V, curves (a)–(e), in the presence of a fixed concentration of glucose, 50 mM, and variable concentrations of dopamine, yet in the absence of added H_2_O_2_. For comparison, the rates of oxidation of dopamine in the presence of the **(2a)** nucleic acid-modified pA-AuNPs, where (**2a)** includes the scrambled sequence of the DBA conjugated to the pA sequence-stabilized AuNPs, and the oxidation rates of dopamine by the separated pA-stabilized AuNPs and the DBA, in the presence of glucose, are presented in curves (f) and (g), respectively. All aptananozyme bioreactors stimulate the glucose-driven aerobic catalytic cascade, leading to the formation of aminochrome. While the cascaded oxidation of dopamine in the presence of glucose and the bare pA-AuNPs is inefficient, curve (g), effective glucose-driven oxidation of dopamine in the presence of all aptananozyme assemblies proceeds, following the order aptananozyme I < aptananozyme II < aptananozyme III < aptananozyme IV. The aptananozyme V, consisting of the 12 spacers bridges separating the dopamine aptamer from pA stabilizing AuNPs, reveals the lowest cascaded activity among the aptananozyme assemblies. The aptananozyme IV, consisting of the 8 spacers separating the dopamine aptamer from pA stabilizing the AuNPs, reveals a *ca*. 13-fold enhanced cascade activity as compared to the bare pA-AuNPs and the separated DBA, curve (d) *vs*. curve (g). Interestingly, the rates of aerobic oxidation of glucose by the **(4)**-pA-AuNPs and the non-aptamer-conjugated pA-AuNPs (in the presence of the separated DBA) to form gluconic acid and H_2_O_2_ are almost similar, Figure S9. Thus, effective oxidation of dopamine by the (**4)**-stabilized pA-AuNPs (and all other aptananozymes) can be attributed to the dopamine aptamer linked to the aptananozyme bioreactor assembly. That is, for the separated pA-AuNPs/DBA system, the concentration of dopamine near the nanozyme interface is low. As a result, the H_2_O_2_ formed by the aerobic oxidation of glucose escapes to the bulk solution, leading to inefficient diffusional oxidation of dopamine. In turn, the concentration of dopamine by the aptananozyme catalyst tethered with aptamer unit (**4)** (or other aptamer structures) leads to effective utilization of the H_2_O_2_ generated by the aptananozyme-catalyzed aerobic oxidation of glucose, resulting in the efficient cascaded oxidation of dopamine, forming aminochrome. That is, the formation of H_2_O_2_ at the DBA-functionalized pA-AuNPs interface yields a high local concentration of H_2_O_2_ for the sequestered, cascaded oxidation of dopamine.^44^ (For further discussion on the significance of the DBA-modified pA-AuNPs aptananozyme to drive the cascaded oxidation of dopamine through the aerobic oxidation of glucose, see Figure S10 and the accompanying discussion.) The efficiencies of the catalyzed glucose-driven aerobic oxidation of dopamine to aminochrome by aptananozymes follow the order I < II < III < IV and fit well with the *K*_d_ values of different aptananozymes toward dopamine and their efficacies to concentrate dopamine at the bioreactor interface.

**Table 2:** Kinetic Parameters Associated with the Set of Aptananozymes I–V and Control Systems, and Accompanying Dissociation Constants of the Respective Dopamine and Aptananozymes^a^

aptananozymes	*V*_max_ (μM min^–1^)	*K*_m_ (μM)	*k*_cat_ (s^–1^)
IV	0.50 ± 0.02	71 ± 11	1.7 ± 0.20
III	0.42 ± 0.02	72 ± 17	1.4 ± 0.15
II	0.38 ± 0.01	76 ± 12	1.3 ± 0.13
I	0.31 ± 0.02	77 ± 13	1.0 ± 0.08
V	0.25 ± 0.02	90 ± 20	0.8 ± 0.09
scrambled DBA/pA-AuNPs	0.12 ± 0.01	113 ± 32	0.4 ± 0.06
separated DBA/pA-AuNPs^b^	0.038 ± 0.06	388 ± 52	0.1 ± 0.01

a All experiments were performed in a 5 mM MES buffer solution, pH 5.5, that included 5 mM MgCl_2_, 100 mM NaCl, and 5 nM of the respective aptananozymes or control system and 5 mM H_2_O_2_. Error bars derived from *N* = 3 experiments.

b The separated pA-AuNPs/DBA system shows pseudo-first-order kinetics: *k* = 0.1 s^–1^.

**Figure 4:**
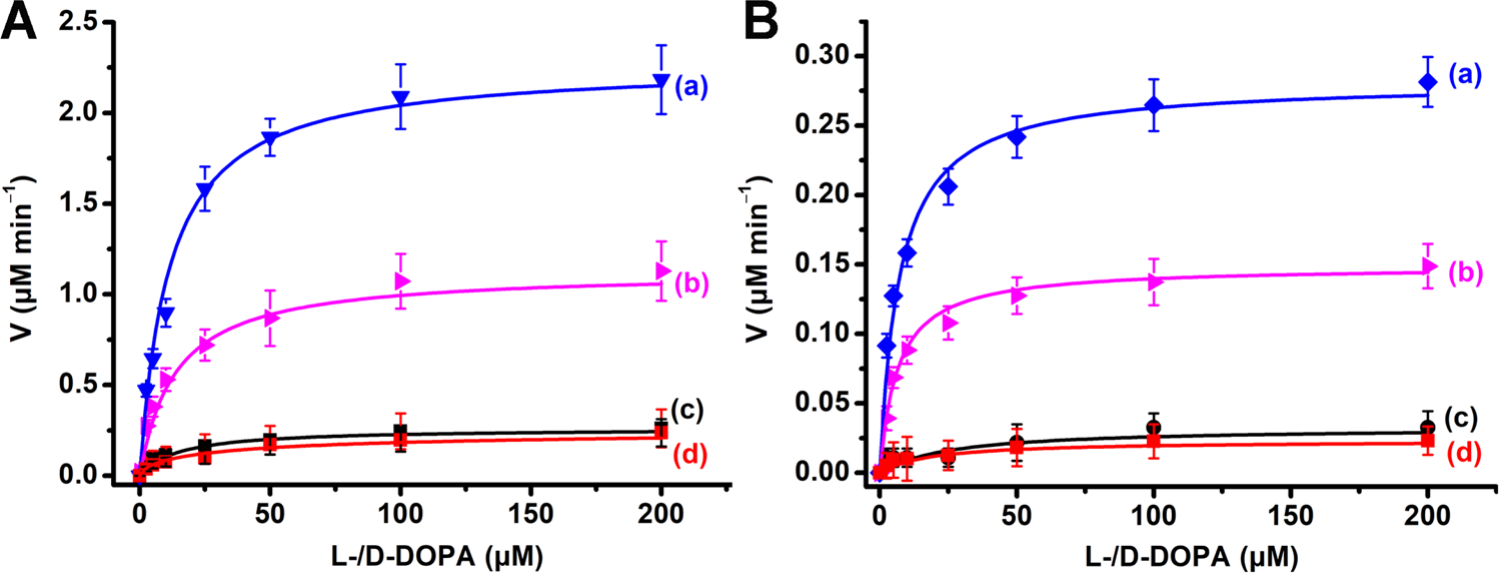
(A) Rates corresponding to the **(4)**-pA-AuNPs (aptananozyme IV)-catalyzed oxidation of l-DOPA (a) and d-DOPA (b) by H_2_O_2_, 10 mM, to yield l-dopachrome and d-dopachrome in the presence of variable concentrations of l-DOPA and d-DOPA, respectively. (c, d) Rates corresponding to the oxidation of l-DOPA and d-DOPA in the presence of variable concentrations of l-/d-DOPA by H_2_O_2_, using the separated pA-AuNPs and DBA, respectively. (B) Rates corresponding to the aptananozyme IV-catalyzed oxidation of l-DOPA (a) and d-DOPA (b) by H_2_O_2_ generated by the aerobic oxidation of glucose, 50 mM, to yield l-dopachrome and d-dopachrome in the presence of variable concentrations of l-DOPA and d-DOPA, respectively. (c, d) Rates corresponding to the oxidation of l-DOPA and d-DOPA in the presence of variable concentrations of l-/d-DOPA by H_2_O_2_ generated by the aerobic oxidation of glucose, 50 mM, using the separated pA-AuNPs and DBA, respectively. In all experiments, the concentration of the aptananozyme IV corresponds to 5 nM. Error bars are derived from *N* = 3 experiments.

The chiral features of the dopamine aptamer suggest that the diastereomeric interactions of l-DOPA or d-DOPA upon binding to the chiral aptamer would yield aptamer–ligand complexes of different binding affinities. Accordingly, the concentration of l-DOPA or d-DOPA at the (**4)**-modified pA-AuNPs will be dictated by the affinities of diastereomeric ligand–aptamer complexes, and thus, chiroselective oxidation of l-/d-DOPA should proceed. Figure 4A shows the oxidation rates of l-DOPA, curve (a), and d-DOPA, curve (b), by H_2_O_2_, 20 mM, in the presence of (**4)**-functionalized pA-AuNPs. For comparison, curves (c) and (d) show the rates of oxidation of l-/d-DOPA by the non-aptamer pA-stabilized AuNPs. The (**4**)-modified pA-AuNPs, aptananozyme IV, reveals chiroselective oxidation of the DOPA ligands, and the rates of oxidation of l-DOPA are *ca*. 2.1-fold enhanced as compared to the rates of oxidation of d-DOPA, Figure 4A. The chiroselective oxidation of the DOPA ligands is consistent with the binding affinities of the DOPA ligands to the (**4**)-aptamer-binding site (*K*_d_ of l-DOPA and d-DOPA to the **(4)**-modified pA-AuNPs corresponding to 4.2 μM and 11.3 μM, respectively, Figure S11). That is, the higher binding affinity of l-DOPA to the aptamer site improves the concentration of the ligand at the catalytic interface, resulting in the chiroselective oxidation. In turn, the polyA-non-aptamer-modified AuNPs do not show any chiral discrimination toward the oxidation of l-/d-DOPA, Figure 4A curves (c) and (d). Despite the chiral properties of the pA interface stabilizing the AuNPs, no chiroselective oxidation of the DOPA substrates is observed. Presumably, the discriminative stereoisomeric interactions of l-/d-DOPA by the chiral interface are too weak to induce any detectable chiroselective oxidation. The chiroselective oxidation ofl-/d-DOPA by H_2_O_2_ to dopachrome was, then, extended to include the chiroselective oxidation of l-/d-DOPA by applying the pA-AuNPs-DBA aptananozyme bioreactor system to catalyze the aerobic oxidation of glucose as the H_2_O_2_ source. Figure 4B depicts the rates of chiroselective oxidation of l-/d-DOPA by the glucose and aptananozyme bioreactor in the presence of variable concentrations of l-/d- DOPA. The oxidation of l-DOPA, curve (a), is two-fold enhanced as compared to the rate of oxidation of d-DOPA, curve (b). For comparison, the rates of oxidation of the l-/d-DOPA, at different concentrations of l-/d-DOPA, by the separated pA-AuNPs nanozyme reactor and the diffusional dopamine aptamer are displayed in curves (c) and (d). The rates of l-/d-DOPA oxidation by the separated glucose/nanozyme reactor are very low, and no chiroselectivity is detectable. The enhanced oxidation rates of l-/d-oxidation are attributed to concentration of the l-/d-DOPA at the catalytic aptananozymes reactor interface by means of the aptamer constituents. The chiroselectivity follows the enhanced binding affinity of l-DOPA, as compared to d-DOPA, to the chiral aptamer units.

An important aspect involves addressing the mechanistic path by which the DBA-functionalized pA-AuNPs, i.e., the aptananozymes, catalyze the oxidation of the different catechol derivatives by H_2_O_2_ or upon interaction with glucose under aerobic conditions. Electron paramagnetic resonance (EPR) experiments confirm that subjecting the aptananozymes to H_2_O_2_ yields hydroxyl radicals, Figure S12A, as ROS. Similarly, treatment of the aptananozyme with glucose under aerobic conditions leads to the detection of the hydroxyl radical, ^•^OH, as ROS, Figure S12B. Control experiments indicated that treatment of the aptananozyme with glucose, under nitrogen, did not lead to hydroxyl radicals, Figure S12C. These results are consistent with the supporting experiments that indicated the aptananozyme catalyzed aerobic oxidation of glucose to gluconic acid and H_2_O_2_. The resulting H_2_O_2_ provides, then, the source for ^•^OH. That is, the formation of hydroxyl radicals as the reactive species in the catalyzed oxidation of the catechol substrates by H_2_O_2_, or the aptananozyme-catalyzed oxidation of the catechol substrates in the presence of glucose, under aerobic conditions, is confirmed. Furthermore, addition of dopamine to the mixture of H_2_O_2_ and the aptananozyme or the mixture of glucose and the aptananozyme led to the depletion of the ^•^OH in the systems, Figure S12D,E, implying that the ^•^OH is consumed by dopamine. Accordingly, and in view of a detailed kinetic study examining the reaction of ^•^OH with catechol derivatives,^76^ a possible mechanism for the ^•^OH-driven formation of aminochrome is provided in Figure S13. Along this path, the ^•^OH-driven oxidation of the catechol substrates yields a geminal ortho bis-phenoxy biradical that is a canonic form of the *o*-benzoquinone state. The intramolecular 1,4-Michael addition within the ortho quinone products of dopamine or l-/d-DOPA yields the aminochrome or dopachrome products.

**Figure 5:**
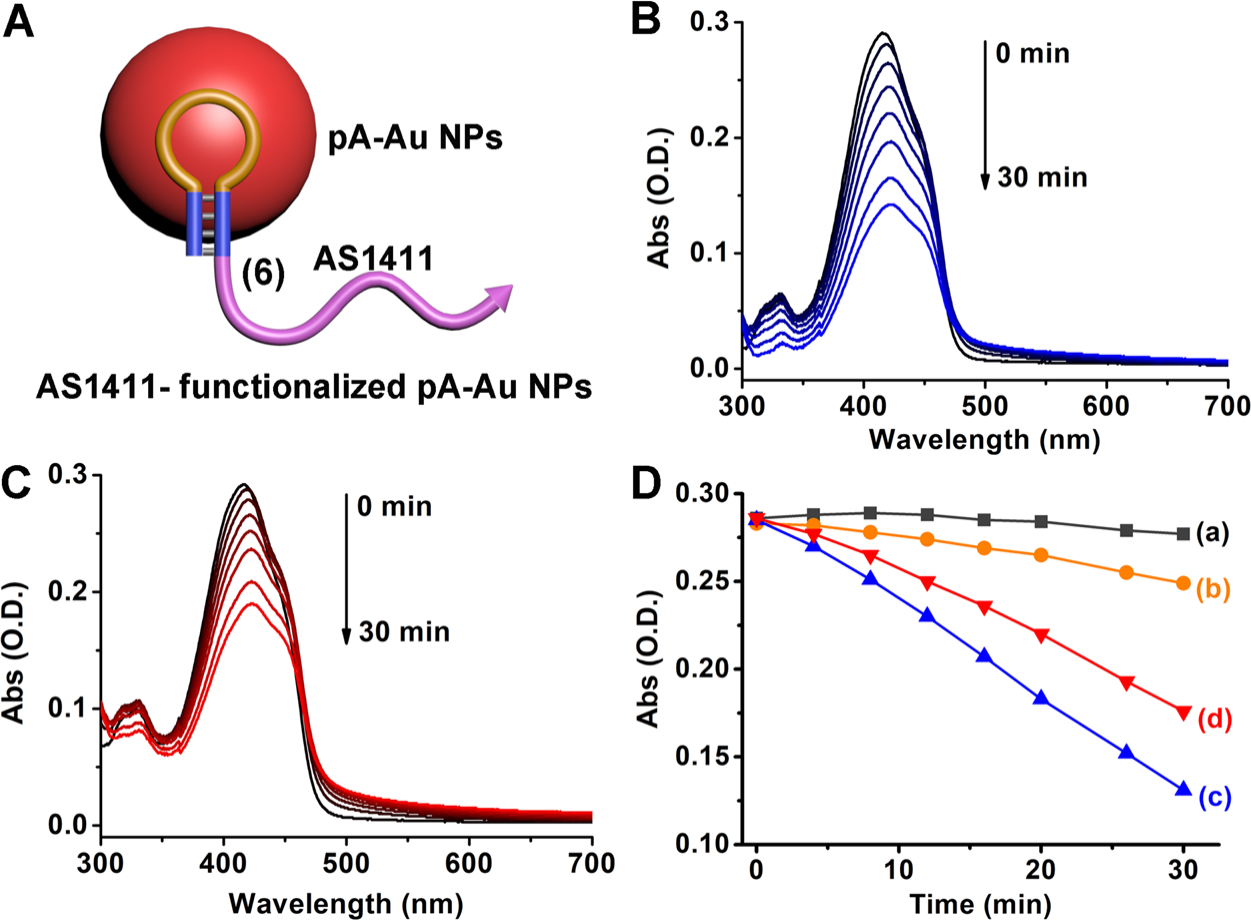
(A) Schematic configuration of AS1411-conjugated pA-AuNPs. (B) Time-dependent absorbance spectra of DPBF upon reaction with the ROS species generated by the AS1411-functionalized pA-AuNPs in the presence of H_2_O_2_, and (C) the AS1411-functionalized pA-AuNPs in the presence of glucose under aerobic conditions. (D) time-dependent absorbance changes of ROS agents (λ = 410 nm) in the systems (a) in the absence of H_2_O_2_, (b) in the presence of H_2_O_2_, (c) composed of the AS1411-functionalized pA-AuNPs in the presence of H_2_O_2_, and (d) composed of the AS1411-functionalized pA-AuNPs in the presence of glucose under aerobic conditions.

Recent studies demonstrated that ROS generated by nanozymes act as cytotoxic agents against cancer cells, and thus these catalytic nanostructures may act as chemodynamic agents for cancer therapy.^77,78^ The limitations associated with the use of nanozymes for chemodynamic treatment of cancer cells include, however, poor cellular permeation and insufficient selective discrimination between cancer cells and normal cells. The discovery that pA-AuNPs catalyze the aerobic oxidation of glucose (present in cancer cells) to form gluconic acid and H_2_O_2_, while generating intermediate ROS products, suggested that the pA-AuNPs could act as reactive nanozymes for chemodynamic treatment of cancer cells. However, to overcome the difficulties associated with nanozymes for chemodynamic treatment of cancer cells, we decided to conjugate the AS1411 aptamer, that binds to the nucleolin receptor associated with different cancer cells,^79,80^ to the pA-AuNPs nanozymes as a means to target the nanozyme to cancer cells and facilitate the permeation of the nanozymes into the cancer cells, thereby enhancing the selectivity and chemodynamic efficacy of the nanozymes. Accordingly, the AS1411 aptamer tethered to the pA-hairpin, (**6)**, was used to stabilize the formation of single hybrid AS1411 aptamer–pA-AuNPs, Figure 5A. The resulting nanozymes reveal the capacity to generate ROS species in the presence of H_2_O_2_ or upon treatment with glucose under aerobic conditions. The formation of the ROS species was confirmed by the reported assay^81^ that followed the depletion of the absorbance of 1,3-diphenyl-isobenzofuran (DPBF) upon reaction with the ROS products. Figure 5B depicts the temporal depletion of the DPBF absorbance spectra in the presence of H_2_O_2_ and the (**6**)-pA-AuNPs nanozyme, and Figure 5C shows the temporal depletion of the absorbance of DPBF upon the (**6)**-pA-AuNPs nanozyme-catalyzed oxidation of glucose under aerobic conditions. Figure 5D summarizes the time-dependent absorbance changes of DPBF upon treatment of the ROS probe with H_2_O_2_ or O_2_/glucose in the presence or absence of the (**6**)-AuNPs. (For the control systems, see Figure S14.) The results indicate that the nanozyme is, indeed, essential to generate the ROS intermediates.

**Figure 6:**
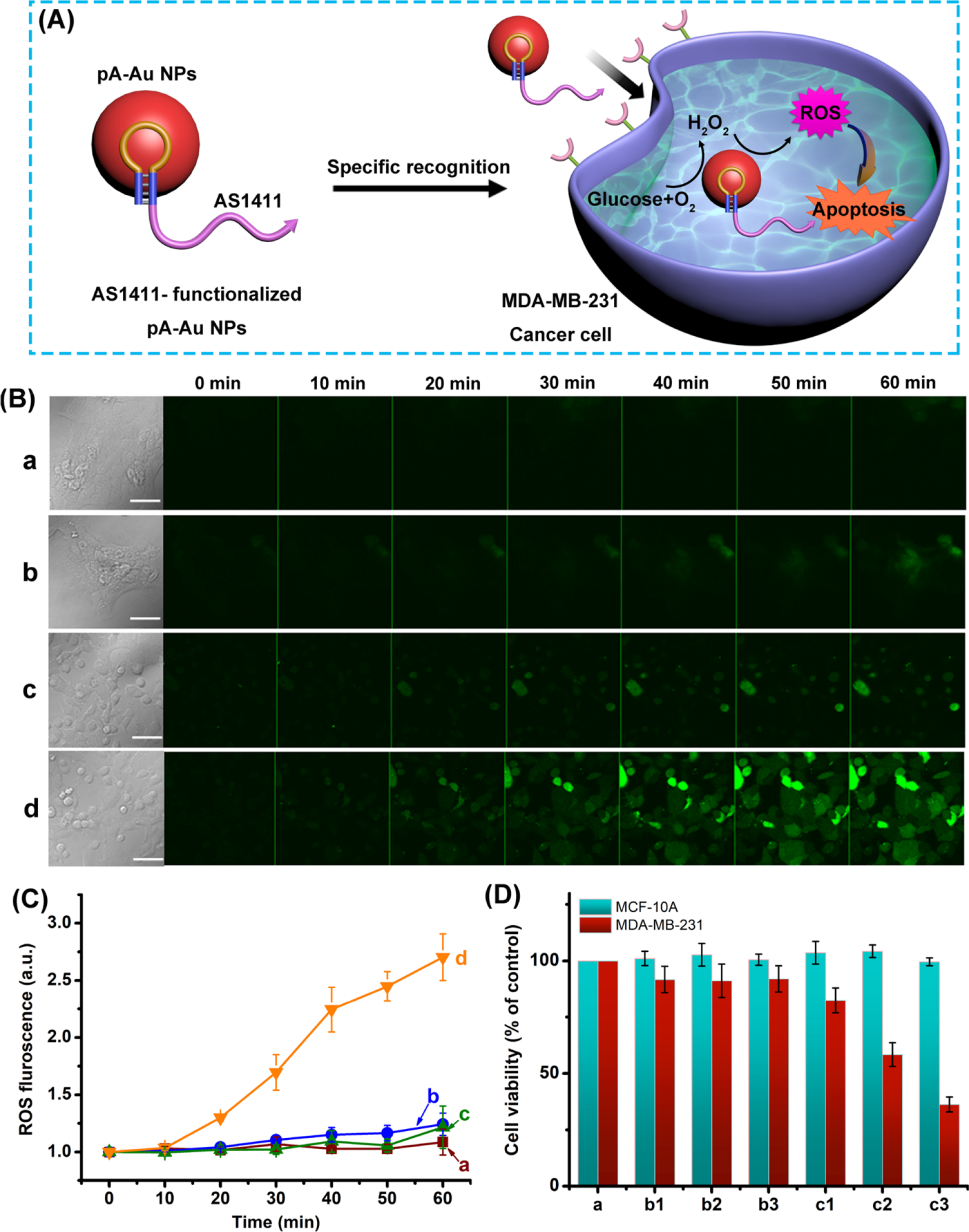
(A) Schematic presentation of the chemodynamic treatment of an MDA-MB-231 cancer cell using targeting AS1411 aptamer-functionalized pA-AuNPs, a ROS-generating nanozyme. (B) Temporal imaging of ROS intermediates generated in (a) epithelial MCF-10A breast cells treated with pA-AuNPs (lacking the AS1411 aptamer), (b) epithelial MCF-10A breast cells treated with the AS1411 aptamer conjugated to the pA-AuNPs, (c) MDA-MB-231 breast cancer cells treated with the pA-AuNPs (lacking the AS1411 aptamer units), and (d) MDA-MB-231 breast cancer cells treated with the AS1411 aptamer conjugated to the pA-AuNPs. In all experiments, cells were treated with 5 nM of the respective NPs. (C) Time-dependent integrated fluorescence intensities of ROS products generated by (a) MCF-10A cells treated with pA-AuNPs, (b) MCF-10A cells treated with AS1411/pA AuNPs conjugate, (c) MDA-MB-231 cancer cells treated with pA-AuNPs, and (d) MDA-MB-231 cancer cells treated with AS1411/pA-AuNPs conjugate. Fluorescence was generated by staining the respective cells with di(acetoxymethyl ester)-6-carboxy-2′,7′-dichlorodihydrofluorescein diacetate (C-DCDHF-DA). Error bars derived by analyzing *N* = 4 frames of cells. (D) Cell viability of MCF-10A epithelial breast cells (green columns) and MDA-MB-231 breast cancer cells (red columns) treated with (a) control system consisting of untreated cells; columns (b1), (b2), and (b3) correspond to treatment of the cells for 2 days with pA-AuNPs (non-aptamer conjugates) using doses corresponding to 0.9 nM, 1.2 nM, and 1.5 nM, respectively, and columns (c1), (c2), and (c3) correspond to cells treated with the AS1411/pA-AuNPs conjugates using doses corresponding to 0.9 nM, 1.2 nM, and 1.5 nM.

The capacity of the AS1411 aptamer/pA-AuNPs conjugate to generate ROS products was, then, applied to examine the use of the NPs as intracellular agents for targeted chemodynamic treatment of cancer cells. In these experiments, MDA-MB-231 breast cancer cells and MCF-10A epithelial breast cells were treated with the AS1411 aptamer/pA-AuNPs with the aim to probe the potential selective cytotoxicity of the NPs toward cancer cells that are associated with the nucleolin receptor. As a result, the ROS generation capacity revealed by the aerobic catalyzed oxidation of intracellular glucose, Figure 6A. The time-dependent formation of the ROS products in the different cells is displayed in Figure 6B,C. The confocal microscopy images shown in Figure 6B display the temporal formation of the ROS products in MCF-10A cells treated with pA-AuNPs (lacking the aptamer conjugate) and the AS1411-conjugated pA-AuNPs, run (a) and run (b), and in the MDA-MB-231 cancer cells treated with the pA-AuNPs and AS1411 aptamer-conjugated pA-AuNPs, run (c) and run (d), respectively, upon staining the cells with di(acetoxymethyl ester)-6-carboxy-2′,7′-dichlorodihydrofluorescein diacetate (C-DCDHF-DA), a ROS indicator dye (λ_ex_ = 488 nm; λ_em_ = 517 nm). Figure 6C shows the integrated time-dependent fluorescent intensities of the confocal frames (error bars derived from *N* = 4 frames at each time interval). Evidently, only the MDA-MB-231 cancer cells treated with the AS1411-conjugated pA-AuNPs demonstrate the accumulation of the ROS products, Figure 6B, run (d), and Figure 6C, curve (d). These results are consistent with the fact that selective aptamer-assisted permeation of the AS1411/pA-AuNPs proceeds only in the MDA-MB-231 breast cancer cells that include the nucleolin receptor, resulting in the selective O_2_/glucose-driven catalyzed generation of the ROS products. The selective chemodynamic treatment of the MDA-MB-231 cancer cells by the ROS agents is demonstrated in Figure 6D. In these experiments, the MDA-MB-231 breast cancer cells and the MCF-10A epithelial breast cancer cells were treated with different doses of the AS1411-conjugated pA-AuNPs, runs (c1)–(c3), and compared to control systems, where the cell lines were treated with different doses of “bare” (non-aptamer-functionalized) pA-AuNPs, runs (b1)–(b3). Evidently, the pA-AuNPs lacking aptamer have no cytotoxic effect on both cell lines due to inefficient permeation into the cells. In turn, the AS1411-conjugated pA-AuNPs reveal selective chemodynamic cytotoxicity toward the MDA-MB-231 breast cancer cells that is enhanced as the dose of the NPs increases. After 2 days of treatment of the MDA-MB-231 breast cancer cells with AS1411-pA-AuNPs, dose of 1.5 nM, *ca*. 70% of cell death was observed. Using a series of concentrations of the AS1411-pA-AuNPs probing the cytotoxicity toward MDA-MB-231 breast cancer cells, Figure S15, the IC_50_ value of the aptananozyme was estimated to be *ca*. 1.27 nM. The results are consistent with the selective aptamer-assisted permeation of the AS1411/pA-AuNPs into the cancer cells.

**Figure 7:**
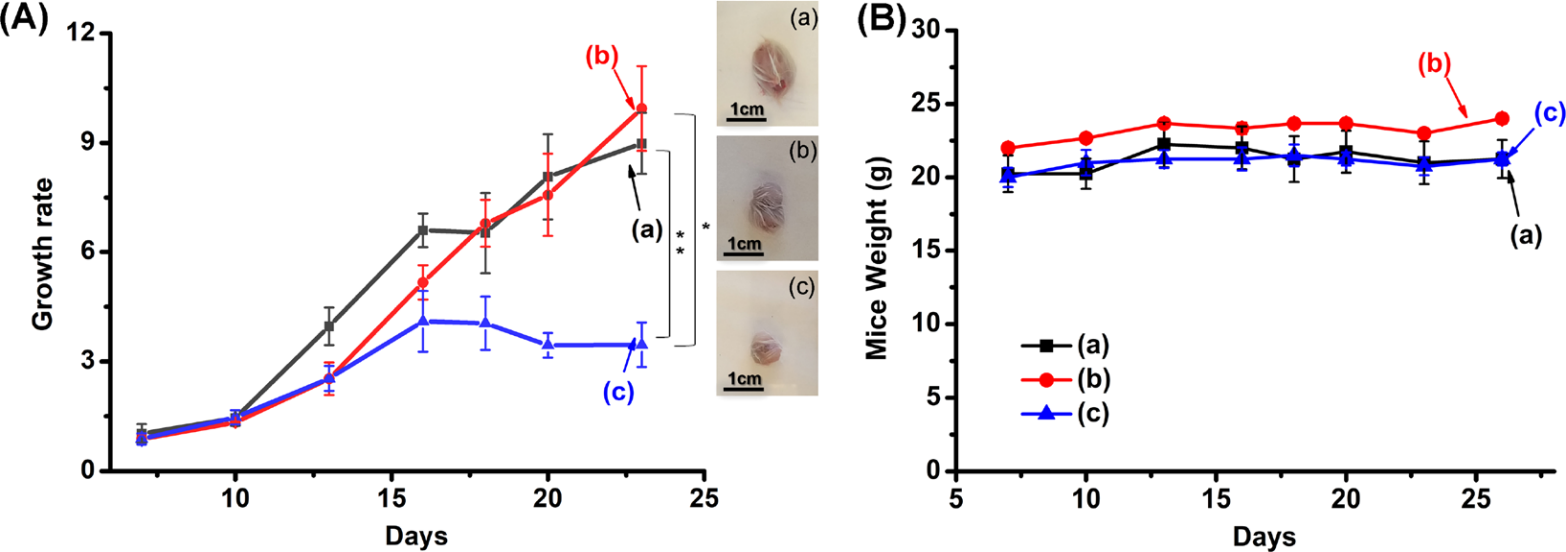
(A) Growth rate profile of the MDA-MB-231 tumors treated with (a) r-AS1411/pA-AuNPs, (b) pA-AuNPs, and (c) AS1411/pA-AuNPs. Inset: images of the extruded tumors grown after treatment with the respective nanoparticles. (B) Weight changes of the MDA-MB-231 tumor-bearing mice upon the treatment with (a) r-AS1411/pA-AuNPs, (b) pA-AuNPs, and (c) AS1411/pA-AuNPs. All results are presented as mean ± SEM. Significant results were evaluated using a *t* test; *P < 0.05, **P < 0.01.

The chemodynamic cytotoxicity of the AS1411/pA-AuNPs toward MDA-MB-231 breast cancer cells was, then, followed by preliminary *in vivo* experiments, Figure 7. In these experiments, xenograft epithelial MDA-MB-231 breast cancer tumors were developed in NOD-SCID mice, and the resulting tumors (80–100 mm^3^) were subjected to intra-tumoral (IT) injection of the AS1411/pA-AuNPs or control systems consisting of pA-stabilized AuNPs, lacking the aptamer tether, pA-AuNPs, or pA-stabilized AuNPs conjugated to a randomized base sequence of the AS1411 aptamer, r-AS1411/pA-AuNPs. The tumor-bearing mice were subjected to a total number of seven injections at time intervals of 2–3 days. (For experimental details, see the Supporting Information.) Figure 7A depicts the average time-dependent volume changes of the tumors in the different mice samples. While a higher growth rate of the tumors treated with the r-AS1411/pA-AuNPs or the bare pA-AuNPs was observed, Figure 7A, curves (a) and (b), the growth rate of the MDA-MB-231 xenograft tumors treated with the AS1411/pA-AuNPs (curve c) was significantly dampened, and after a time-interval of ca. 23 days, the sizes of the tumors were almost identical to the original values. These results are consistent with the effective permeation of the AS1411/pA-AuNPs into the MDA-MB-231 breast cancer cells and their effective chemodynamic intracellular generation of ^•^OH as toxic ROS agent. Furthermore, Figure 7B shows the weight changes of the mice treated with the different pA-AuNPs systems. No weight losses of the mice were observed during the treatment with the respective nanoparticles, indicating non-toxic effects of the particles toward the mice.

## Conclusions

This study has expanded the scope of aptamer-modified nanozymes (aptananozymes) to a system performing simultaneous and cascaded catalysis. The system involved the pA-AuNPs-catalyzed aerobic oxidation of glucose to gluconic acid and H_2_O_2_ that is coupled to the effective hydrogen peroxide-catalyzed oxidation of dopamine (or l-/d- DOPA) to aminochrome (or l-/d-dopachrome). The aptamer strands linked to the nanozyme particles provide binding sites for the association of the catechol substates in spatial proximity to the catalyst. Concentration of the substrates at the catalytic surface resulted in effective oxidation of the substrate and provided chiral receptor binding sites for the chiroselective oxidation of l-/d-DOPA to l-/d-dopachrome. The binding modes of the aptamers and the strands’ flexibilities dominated by spacer bridges affect the binding properties of the aptamers and the resulting enhanced catalytic activities of the aptananozymes. An important result of the study is, however, the demonstration that a single nanoparticle stimulates simultaneously two different catalytic processes. This feature of the aptananozyme is rarely observed for native enzymes that catalyze a single target or chemical transformation. The dual catalytic activities of the artificial aptananozyme enabled a cascaded catalytic transformation within the confined structure of the aptananozyme that acted as a bioreactor. The mechanistic analysis of the aerobic oxidation of the catechol substrates by glucose demonstrated the participation of ROS intermediates in the reactions.

The capacity of the pA-AuNPs to generate ROS products by the aerobic oxidation of glucose was further applied to tailor aptamer-functionalized pA-AuNPs conjugates for the chemodynamic treatment of cancer cells. In these experiments, the AS1411 aptamer, recognizing the nucleolin receptor associated with different cancer cells, was conjugated to the pA-AuNPs nanozyme, to yield a functional selective nanozyme catalyst for the chemodynamic treatment of cancer cells, using the aerobic oxidation of glucose as the source for the intracellular formation of ROS products. The selective permeation and chemodynamic treatment of MDA-MB-231 cancer cells, as compared to MCF-10A epithelial cells, was demonstrated. In addition, preliminary *in vivo* experiments in MDA-MB-231 xenograft tumor-bearing mice revealed the high chemodynamic cytotoxic efficacy of the AS1411/pA-AuNPs. The functionalization of the pA-AuNPs with sequence-specific aptamers, and their capacity to yield ROS products through the aerobic oxidation of glucose, makes it possible to design functional pA-AuNPs aptananozymes for other cancer biomarkers, diverse diseases, and antibacterial applications.

## Experimental Section

The sequences of the nucleic acids used in the study are shown here.

**1**: 5′-CGACGCCAGTTTGAAGGTTCGTTCGCAGGTGTGGAGTGACGTCGGTAACAACAGTCAAAAAAAAAAAAAAAAAAAAGACTGTTGTTAC-3′

**2**: 5′-GTAACAACAGTCAAAAAAAAAAAAAAAAAAAAGACTGTTGTTACCGACGCCAGTTTGAAGGTTCGTTCGCAGGTGTGGAGTGACGTCG-3′

**3**: 5′-GTAACAACAGTCAAAAAAAAAAAAAAAAAAAAGACTGTTGTTACTGTACGACGCCAGTTTGAAGGTTCGTTCGCAGGTGTGGAGTGACGTCG-3′

**4**: 5′-GTAACAACAGTCAAAAAAAAAAAAAAAAAAAAGACTGTTGTTACTGTATGTACGACGCCAGTTTGAAGGTTCGTTCGCAGGTGTGGAGTGACGTCG-3′

**5**: 5′-GTAACAACAGTCAAAAAAAAAAAAAAAAAAAAGACTGTTGTTACTGTATGTATGTACGACGCCAGTTTGAAGGTTCGTTCGCAGGTGTGGAGTGACGTCG-3′

**6**: 5′-GTAACAACAGTCAAAAAAAAAAAAAAAAAAAAGACTGTTGTTACGGTGGTGGTGGTTGTGGTGGTGGTGGTTT-3′

**2a**: 5′-GTAACAACAGTCAAAAAAAAAAAAAAAAAAAAGACTGTTGTTACGACTAGCGTGTGTGATGGGACCTTAGGCCGTCACGGGGCTTAGT-3′

**DBA**: 5′-CGACGCCAGTTTGAAGGTTCGTTCGCAGGTGTGGAGTGACGTCG-3′

**pA-strand**: 5′-GTAACAACAGTCAAAAAAAAAAAAAAAAAAAAGACTGTTGTTAC-3′

The detailed procedure to prepare the different aptananozymes, their structural characterization, and the kinetic experiments to evaluate their catalytic functions of the aptananozymes are detailed in the Supporting Information.

## Supporting Information Available

Detailed experimental procedures, gel electrophoresis results, TEM images, absorption spectra of oxidized dopamine products, ITC measurements, EPR spectra corresponding to hydroxyl radical, and suggested mechanism for the oxidation of dopamine by hydroxyl radicals generated by the aptananozyme (PDF)
