# Supplementary material for: Aptamer-Modified Au Nanoparticles: Functional Nanozyme Bioreactors for Cascaded Catalysis and Catalysts for Chemodynamic Treatment of Cancer Cells
Source: ACS Nano. 2022 Oct 26;16(11):18232–43. doi: 10.1021/acsnano.2c05710 (PMC9706657; doi:10.1021/acsnano.2c05710)
Supplement: Supplementary file 1 — nn2c05710_si_001.pdf [file nn2c05710_si_001.pdf]

Supporting Information

Aptamer-Modified Au Nanoparticles: Functional Nanozyme Bioreactors for Cascaded Catalysis and Catalysts for Chemodynamic Treatment of Cancer Cells

Yu Ouyang[1§], *Michael Fadeev*[1§], *Pu Zhang*[1], *Raanan Carmieli*[2], *Jiang Li*[3,4], *Yang Sung Sohn*[5], *Ola Karmi*[5], *Rachel Nechushtai*[5], *Eli Pikarsky*[6], *Chunhai Fan*[3], *Itamar Willner*[1*]

1. The Institute of Chemistry, The Hebrew University of Jerusalem, Jerusalem 91904, Israel.
2. Department of Chemical Research Support, Weizmann Institute of Science, Rehovot, 76100, Israel.
3. School of Chemistry and Chemical Engineering, Frontiers Science Center for Transformative Molecules and National Center for Translational Medicine, Shanghai Jiao Tong University, Shanghai 200240, China.
4. The Interdisciplinary Research Center, Shanghai Synchrotron Radiation Facility, Zhangjiang Laboratory, Shanghai Advanced Research Institute, Chinese Academy of Sciences, Shanghai 201210, China.
5. Institute of Life Science，The Hebrew University of Jerusalem, Jerusalem 91904, Israel.
6. The Lautenberg Center for Immunology and Cancer Research, IMRIC, The Hebrew University of Jerusalem, Jerusalem 91120, Israel.

[§] Yu Ouyang and Michael Fadeev contributed equally to this work.

Instruments

Absorption spectra were recorded on a UV-2450 spectrophotometer (UV, Shimadzu). Kinetic measurements were performed at 25 °C using a Biotek Synergy H1 microplate reader, equipped with a Biotek dual dispensing unit, and using Corning 3696 96-hald-well plates. Dissociation constants were evaluated using Isothermal titration calorimetry (ITC) instrument (Malvern instruments MicroCal PEAQ-ITC). Fourier-transform infrared spectroscopy (FTIR) measurements were performed using a Nicolet iS50 FTIR Spectrometer. Transmission Electron Microscopy measurement were performed using a Tecnai G2 Spirit TWIN T12 apparatus. Electron paramagnetic resonance (EPR) measurements were carried out at room temperature using a Bruker ELEXYS E500 spectrometer operating at X-band frequencies (9.5 GHz) and a Bruker ER4102ST resonator.

Materials

Gold (III) chloride trihydrate ($HAuCl_4 \cdot 3H_2O$), trisodium citrate dihydrate ($Na_3C_6H_5O_7 \cdot 2H_2O$), hydrogen peroxide ($H_2O_2$; 30 %), dopamine hydrochloride, ascorbic acid, Amplex red, and glucose were purchased from Sigma Aldrich. The series of modified DBA-pA sequences used to prepare the series of DBA-pA Au NPs are included in Table S1. The strands, HPLC pure, were provided by Integrated DNA Technologies.

Preparation of DBA-conjugated poly-adenosine DNA strands-modified Au NPs

The respective DBA-conjugated poly-adenosine DNA strands (**1**)-(**5**) and (**2a**), in 0.1 M sodium phosphate buffer (PBS, 0.1 M of NaCl, 10 mM of Na_2HPO_4 and NaH_2PO_4, pH 7.4) were incubated in 95°C for 5 min and then cooled to room temperature at a rate of 0.2 °C/min. The DNA hairpin scaffolds were prepared following this step. 10 nM of

DNA hairpin scaffolds were then incubated with 0.8 mM $HAuCl_4$ for 5 h, which allowed the polyA-loop domains to associate with the enough $AuCl_4^-$ units. Then, same amount of trisodium citrate in PB buffer containing 500 mM Na^+ (pH = 6.0) was added to mixture DNA hairpin scaffolds solution under shaking continuously with 35°C. After incubating for 3 h, the pA-Au NPs was collected by centrifuge at 12000 g for 20 min and washed with deionized water for three times. Finally, the nanoparticle product was purified by electrophoretic separation on 3% agarose gel, followed by cutting out to appropriate band corresponding to the single modified DBA-pA-Au NPs. The resulting pA-Au NPs (aptananozyme) were achieved after elution by buffer extraction of the DBA-pA-Au NPs from the cut band and their concentration was determined by UV/vis spectroscopy at 521 nm (ε = 2.7 × 10^8 M^{-1} cm^{-1}).

Kinetic measurements with aptananozymes.

Kinetic measurements were performed at 25 °C using a Biotek Synergy H1 microplate reader equipped with a Biotek dual dispensing unit and using Corning 3696 96-well plates. For dopamine oxidation, the aptananozymes (5 nM) were introduced into 5 mM MES buffer, pH 5.5, 5 mM $MgCl_2$, 100 mM NaCl, and 10 μL of a dopamine, solution at variable concentrations which were added to the respective wells. Subsequently, 10 μL of H_2O_2 (final concentration 5 mM) or glucose (final concentration 50 mM) was dispensed into each well, and the absorbance values of the oxidized products (amidochrome, absorbance at 480 nm, ε = 3058 M^{-1} cm^{-1}) were measured in the different wells for a time interval of 30 min. As for the L-DOPA and D-DOPA oxidation process, the aptananozyme IV (5 nM) was dissolved in 5 mM MES buffer, pH 5.5, 5 mM $MgCl_2$, 100 mM NaCl, and 10 μL of DOPA, consisting of variable concentrations which were added to the respective wells. Subsequently, 10 μL of H_2O_2 (final concentration 10 mM) or glucose (final concentration 50 mM) was dispensed into

each well, and the absorbance values of oxidized products (absorbance at 475 nm, ε = 3600 M^{-1} cm^{-1}) were measured in the different wells for a time interval of 30 min.

EPR measurements.

Radical specie such as $^{\cdot}OH$, were detected using the EPR spin trapping technique coupled with a spin trap 3,4-dihydro-2-methyl-1,1-dimethylethyl ester-2H-pyrrole-2-carboxylic acid-1-oxide (BMPO). Typically, the mixtures of H_2O_2 (5 mM) and aptananozyme II (5 nM), and glucose (50 mM) and aptananozyme II (5 nM), were prepared in 5 mM MES buffer, pH 5.5, 5 mM $MgCl_2$, 100 mM NaCl, including BMPO (0.01 M). The EPR measurements were record under the conditions: Centerfield, 3352.00 G; Sweepwidth, 100.0 G; MW Power, 20.02 mW; Modulation Amplitude, 1.00 G; Modulation Frequency, 100.00 kHz; No of points 512; Conversion Time, 40.96 ms; Conversion Time, 40.96 ms.

ITC evaluation of the K_d values of dopamine or L-/D-DOPA bound to DBA-functionalized aptananozymes.

For evaluation of the K_d values of aptananozymes, a stock solution of the respective aptananozyme, 20 µM, in a 5 mM MES buffer pH 5.5, 100 mM NaCl and 5 mM $MgCl_2$ for the DBA-aptananozymes was prepared. Stock solutions of dopamine (250 µM), L-DOPA (1 mM), and D-DOPA (1 mM) in their respective buffers were prepared. The respective aptananozymes were loaded in the sample cell, 280 µL, and the ITC instrument syringe was added with the respective ligand stock solution. The aptananozyme sample was titrated by injecting repeated 2 µL aliquots of the respective ligand into the measurement cell (total 15-19 injections). The heat difference between the measuring and reference cell, upon each injection were evaluated under the following conditions: Cell reference power, 41.9 µW, syringe rotation rate, 750 rpm,

initial delay of 60 seconds before the first injection to ensure equilibration of sample cell, injections spaced at 150-300 seconds to ensure baseline stabilization between measurements. Each binding experiment was repeated $n = 2$. The resulting K_d fitting was performed using the instrument (MicroCal PEAQ-ITC Analysis Software) with the "one set of sites" binding model and free parameters for K_d, Hill coefficient, and ΔH. All fitted K_d curves yielded a Hill coefficient of 0.9~1, implying a 1:1 complex between the ligands and the aptamers.

Cell experiments

Cell culture: Normal breast cells (MCF-10A) were maintained in complete growth medium consisting of 1:1 mixture of Dulbecco's modified Eagle's medium and Ham's F12 medium supplemented with horse serum (5%), epidermal growth factor (20 ng mL^{-1}), cholera toxin (CT, 0.1 μg mg^{-1}), insulin (10 μg mL^{-1}), hydrocortisone (500 ng mL^{-1}), and penicillin/streptomycin (1 unit mL^{-1}). Human breast cancer cells (MDA-MB-231) were grown in RPMI-1640 medium supplemented with 10% FCS, L-glutamine, and antibiotics (Biological Industries). Cells were plated one day prior to the experiment on 96-well plates for cell viability.

Cell viability experiments: Cell viability was assayed after incubation of pA-Au NPs and AS1411/pA-Au NPs in MCF-10A, MDA-MB-231 cells planted at a density of 1.2×10^4 cells per well in 96-well plates. After 6 hours incubation with these NPs, cells were washed intensively with growth medium. After washing, cells were replenished with full medium and further incubated for 3 days. the cell viability was determined after 2 days with the fluorescent redox probe, Presto-Blue. The fluorescence of Presto-Blue was recorded on a plate-reader (Tecan Safire) after 1 h of incubation at 37 °C ($\lambda_{ex} = 560$ nm; $\lambda_{em} = 590$ nm).

ROS production measurement: ROS production in cancer cells compared to normal cells was determined by incubating cells with pA-Au NPs and AS1411/pA-Au NPs for 3 h and then by incubating cells containing pA-Au NPs and AS1411/pA-Au NPs at 37°C with 10 μM of Carboxy-H_2DCFDA-AM in HEPES-buffered saline (HBS) supplemented with 10 mM glucose. This nonfluorescent molecule is readily converted to a green-fluorescent form when the acetate groups are removed by intracellular esterase and oxidation by the activity of ROS within the cell. The conversion of the non-fluorescent probe to the green-fluorescent form was measured for 1 h at 37°C under the confocal microscopy (the Olympus FV3000 confocal laser-scanning microscope) (λ_{ex} = 488 nm; λ_{em} = 517 nm) and all images were analyzed with image J. \

Mice experiment:

Female NOD-SCID mice were used for detecting breast cancer xenograft tumour cell progression of MDA-MB-231 cells which were injected subcutaneously. The experiment was approved by the Authority for Biological and Biomedical Models at the Hebrew University, ethical number is NS-21-16745-4. MDA-MB-231 of 6×10^6 cells/mouse were injected subcutaneously to the flack of each mouse. Tumor mass was generated after 7 days in a volume that is around 80-100 mm^3, then the injections of the treatment were done intra-tumoral (IT) (2-3 times/week) in total 7 injections, by using Control-Au NPs with no aptamer and Control- Au NPs with random Aptamer for the control group, compared to Au-NPs with AS1411 aptamer. All particles were injected in a volume of 100 μl of the amount of 50μg/mouse. NPs were prepared in a final concentration of 1 mg/ml. For each group we used 4 mice. Tumor was majored every 2-3 days before the following injection to evaluate the width and the height, then tumor volume (mm^3) was measured using the equation of ($Width^2 \times Height$)/2, the tumor

growth rate was a ratio of a tumor volume for each reading to the starting volume. Toxicity of the treatment was evaluated by the mice weight change (g) that was measured twice a week. All results were presented as mean ± SEM.

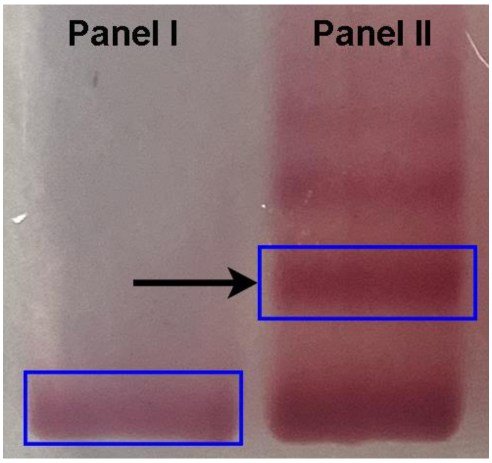

Figure S1. Electrophoretic separation of non-modified Au NPs (panel I) and DBA-poly-adenosine DNA strands (**1**)-modified Au NPs (panel II) by 3% agarose gel. Panel I corresponds to non-modified Au NPs. In panel II, arrow indicates the band corresponding to the DBA-poly-adenosine DNA strands (**1**)-modified Au NPs.

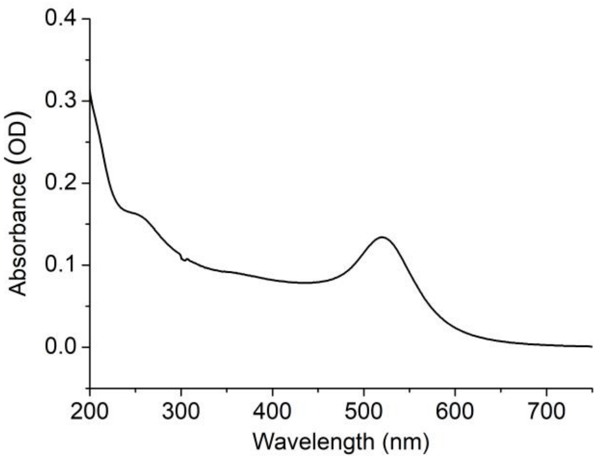

Figure S2. UV spectrum of the aptananozyme (0.5 nM) in reaction buffer (5 mM MES buffer solution, pH 5.5, that included 5 mM $MgCl_2$, 100 mM NaCl). The concentration of 5 nM aptananozyme can be calculated by the absorbance of λ = 521 nm.

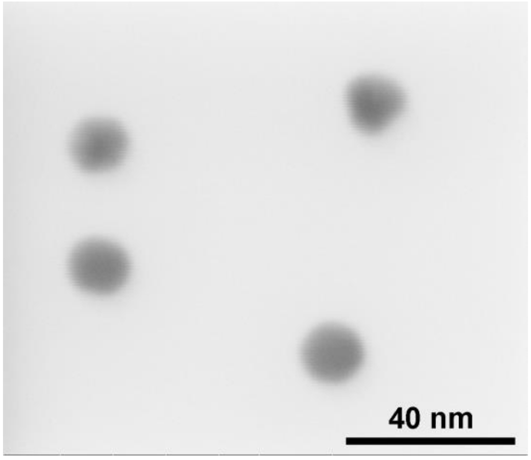

Figure S3. TEM image of the synthesized aptananozyme. The diameter of the Au NPs is *ca.* 13 nm.

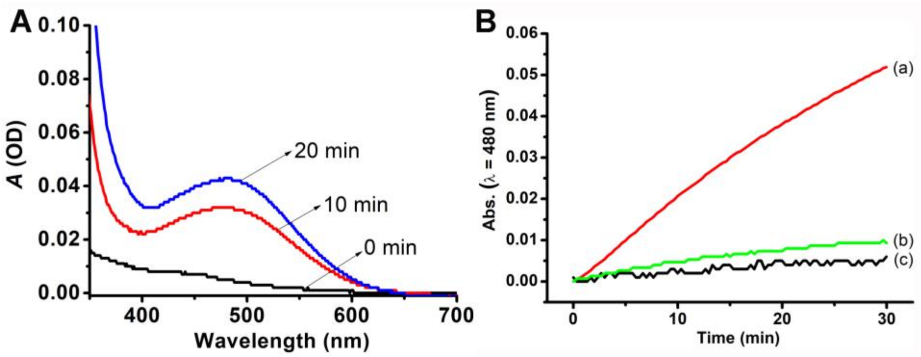

Figure S4. (A) Time-dependent absorption spectra of oxidized dopamine in the presence of 5 nM pA-Au NPs, 5 mM dopamine, and 20 mM H_2O_2. (Aminochrome, λ = 480 nm). (B) Time-dependent absorbance of oxidized dopamine in the in the presence of 5 nM pA-Au NPs (a), in the absence of H_2O_2 (b), and in the absence of pA-Au NPs (c).

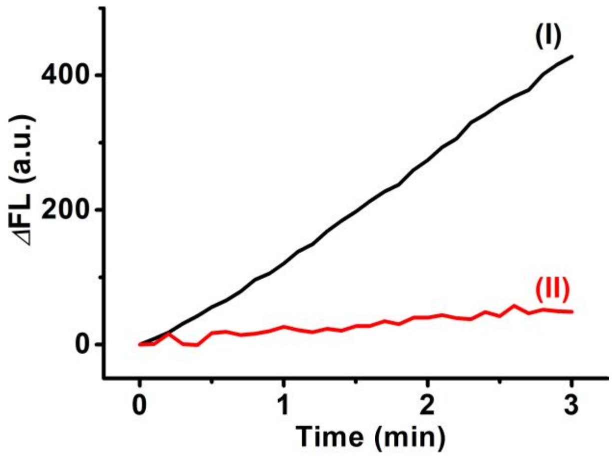

Figure S5. (I) The time-dependent fluorescence changes of Resorufin generated by oxidation of Amplex red in presence of 50 mM glucose,100 nM horseradish peroxidase and 5 nM pA-Au NPs. (II) The time-dependent fluorescence changes of Resorufin generated by oxidation of Amplex Red in presence of 50 mM glucose and 100 nM horseradish peroxidase. (Resorufin, λ_{ex} = 572 nm, λ_{em} = 583 nm)

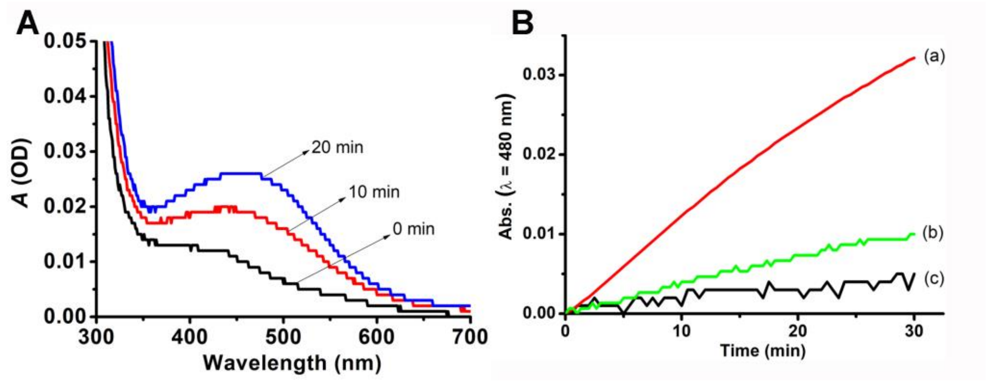

Figure S6. (A) Time-dependent absorption spectra of oxidized dopamine in the presence of 5 nM pA-Au NPs, 10 mM dopamine, and 100 mM glucose. (B) Time-dependent absorbance of oxidized dopamine in the in the presence of 5 nM pA-Au NPs (a), in the absence of glucose (b), and in the absence of pA-Au NPs (c).

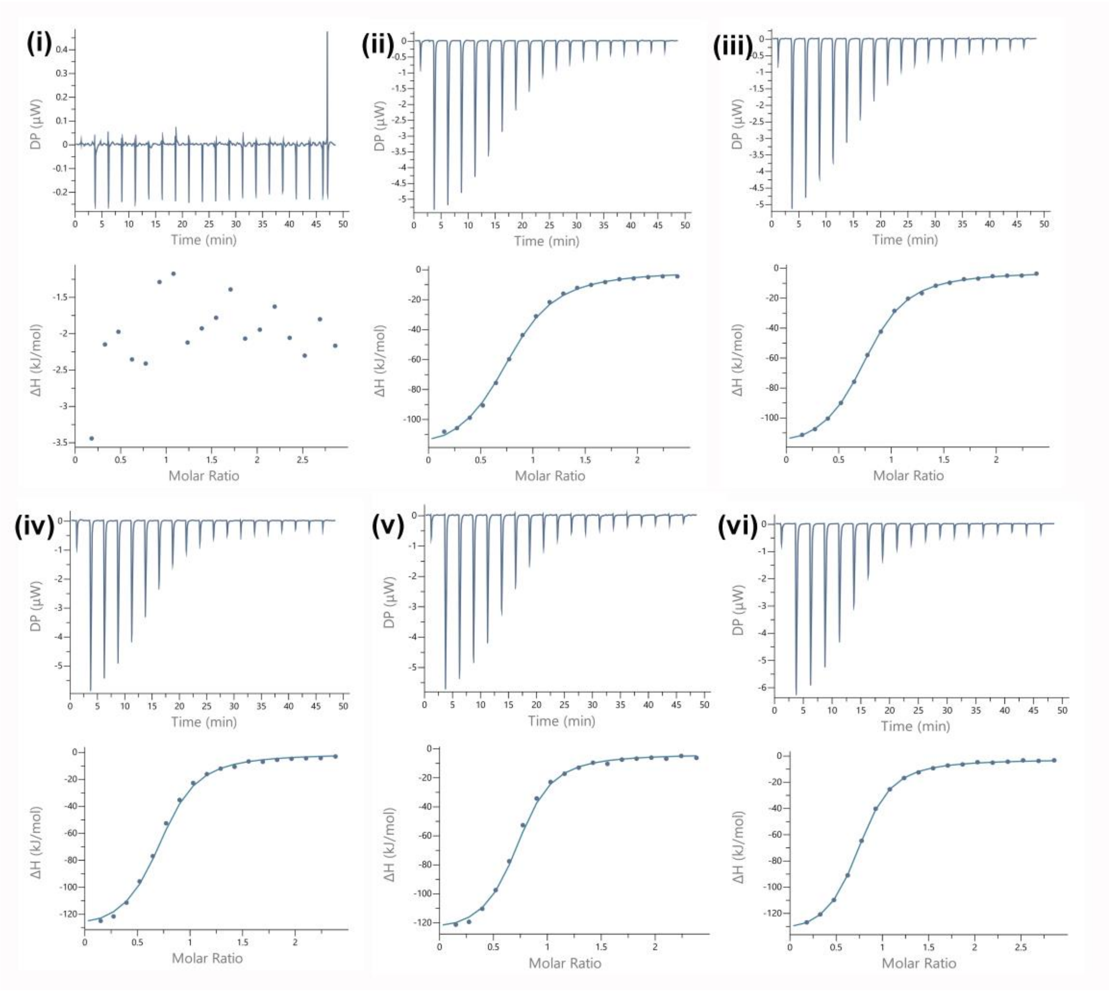

Figure S7. ITC plots (heat flow *vs* time and molar enthalpy change *vs* molar ratio) of different aptananozymes, with dopamine in 5 mM MES pH 5.5, 5 mM $MgCl_2$, 100 mM NaCl: (i) Ce^{4+}-functionalized C-dots with scrambled DBA. (ii) aptananozyme I. (iii) aptananozyme II. (iv) aptananozyme III. (v) aptananozyme IV. (vi) aptananozyme V.

Glucose oxidase-like catalytic activities of the DBA functionalized aptananozyme. The different DBA modified pA-Au nanoparticles, aptananozyme I-V, reveal glucose oxidase-like activities and catalyze the aerobic oxidation of glucose form gluconic acid and H_2O_2, eq.1.

eq.1:

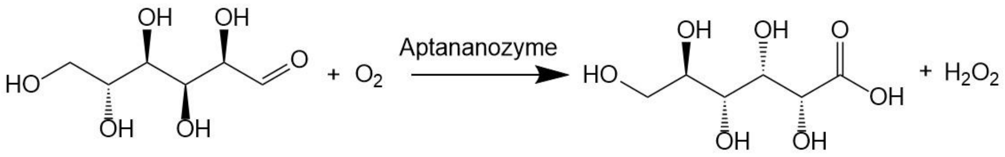

This is exemplified in Figure S8 with the aerobic oxidation of glucose to gluconic acid and H_2O_2. The reaction is probed by monitoring at time intervals the generated H_2O_2 through the fluorescence changes of Resorufin generated by the H_2O_2-stimulated oxidation of Amplex Red (λ_{ex} = 572 nm, λ_{em} = 583 nm), Figure S8A.

eq.2:

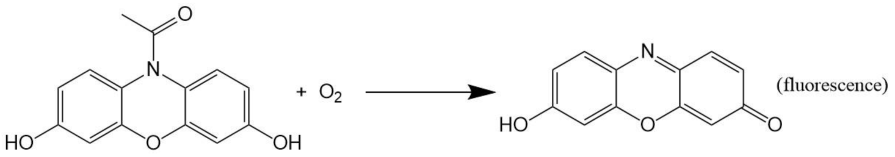

Using a calibration curve corresponding to the fluorescence intensities of Resorufin generated in the presence of known concentrations of H_2O_2, Figure S8B, the time-dependent contents of H_2O_2 generated by the aptananozyme IV catalyzed aerobic oxidation of glucose, eq 1, were evaluated, Figure S8C.

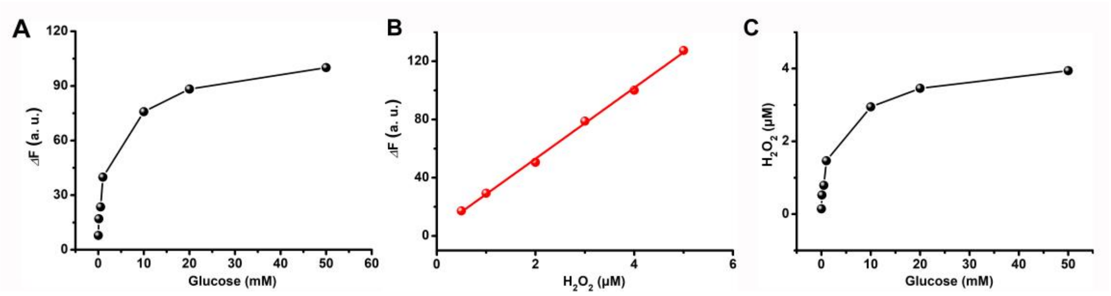

Figure S8. (A) Fluorescence of resorufin produced from oxidation of Amplex Red by variable concentration of glucose in presence of aptananozyme IV and 100 nM horseradish peroxidase with time interval of 3 h. (B) Calibration curve corresponding to the fluorescence intensities of Resorufin generated in the presence of known concentrations of H_2O_2. (C) The concentrations of H_2O_2 generated by aptananozyme under variable concentrations of glucose with time interval of 3 h.

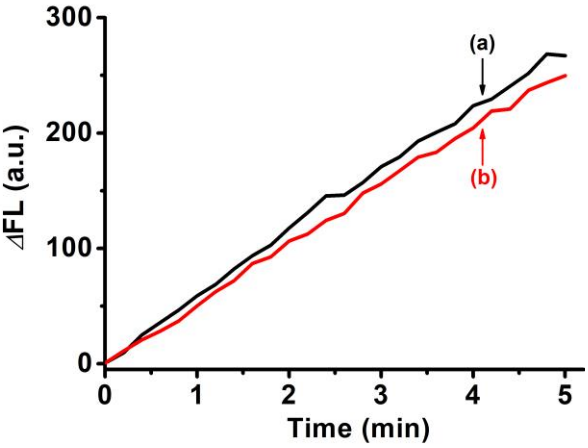

Figure S9. (a) The time-dependent fluorescence of Resorufin generated by oxidation of Amplex red in presence of 50 mM glucose, 100 nM horseradish peroxidase and 5 nM pA-Au NPs. 5 nM (**4**)-functionalized pA-Au NPs. (b) The time-dependent fluorescence of Resorufin generated by oxidation of Amplex red in presence of 20 mM glucose, 100 nM horseradish peroxidase and 5 nM (**4**)-functionalized pA-Au NPs.

Comparison of the aerobic glucose driven oxidation of dopamine to aminochrome by the Aptananozyme IV bioreactor to the analog, glucose oxidase (GOx)/peroxidase (POx), mediated oxidation of dopamine to aminochrome.

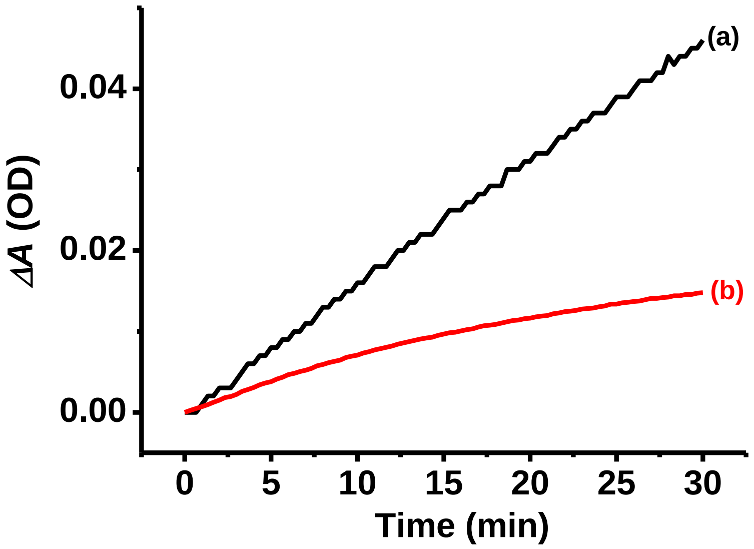

Figure S10. (a) Time-dependent absorption spectra of oxidized dopamine in the presence of aptananozyme IV. (b) Time-dependent absorption spectra of oxidized dopamine in the presence of GOx and POx.

To establish the advantages of the cascaded oxidation of dopamine to aminochrome through the H_2O_2, primary aerobic oxidation of glucose to gluconic acid and H_2O_2, followed by the catalyzed H_2O_2 oxidation of dopamine to aminochrome, using the aptananozyme IV bioreactor, the following control experiment (a random assembly of native glucose oxidase (GOx) and peroxidase (POx) enzymes) was performed. The aptananozyme IV conjugate, 5 nM, was probed toward the aerobic oxidation of glucose, 50 mM, (by following the efficacy of generating H_2O_2 and the subsequent POx catalyzed oxidation of Amplex red to Resorufin).

The GOx activity of the aptananozyme IV was found to be equivalent to a GOx concentration of 1.2 nM. Similarly, the dopamine peroxidase activity only of the

aptananozyme IV was equivalent to a POx concentration of 1.6 nM.

Accordingly, the aptananozyme, 5 nM, were used to probe the aerobic oxidation of dopamine, 1 mM, to aminochrome, in the presence of glucose, 50 mM. The time-dependent absorbance changes associated with the formation of aminochrome are depicted in Figure S10, curve (a). For comparison, the time-dependent absorbance changed corresponding to the oxidation of dopamine, 1 mM, in the presence of glucose, 50 mM, using the native GOx, 1.2 nM, and POx, 1.6 nM (equivalent concentrations to the aptananozyme) are depicted in Figure S10, curve (b). Evidently, the biocatalytic cascade corresponding to the oxidation of dopamine to aminochrome, in the presence of glucose, using the aptananozyme IV bioreactor is *ca.* 3-fold high than the efficacy of dopamine oxidation, in the presence of glucose, using the native GOx/POx system. The enhanced activities of the oxidation of dopamine by the bioreactor system are attributed to the operation of the biocatalytic cascade within the confined environment provided by the aptananozyme hybrid. The aerobic oxidation of glucose by the aptananozyme yields H_2O_2 in close proximity to the dopamine substrate bound to the aptamer constituent, leading to the effective oxidation of dopamine to aminochrome.

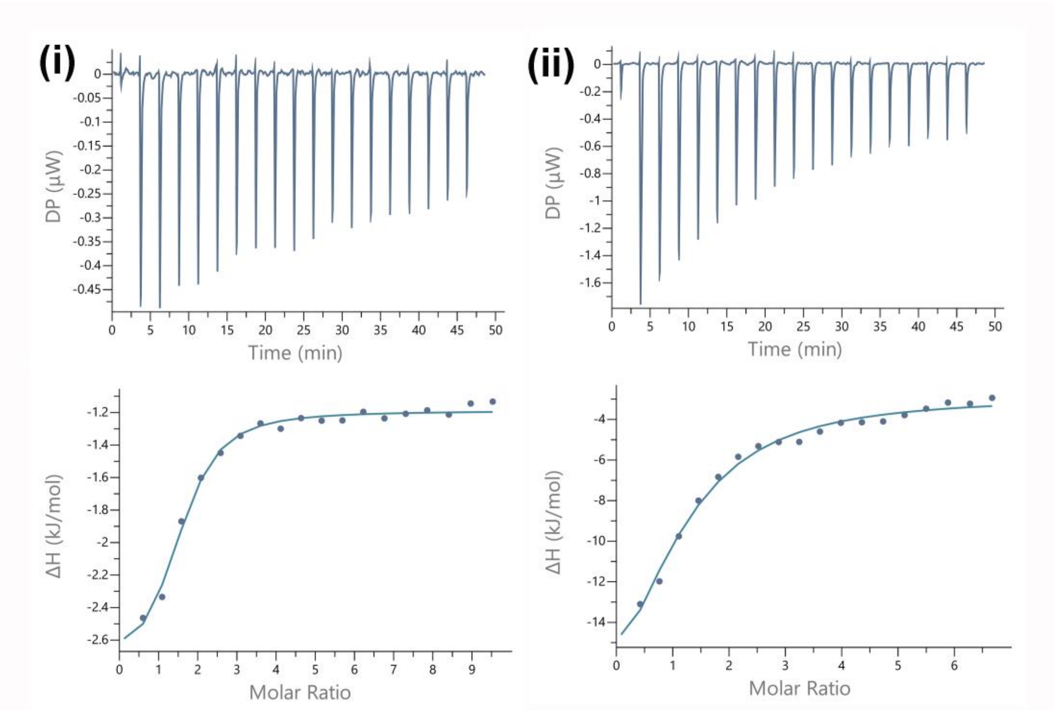

Figure S11. ITC plots (heat flow *vs* time and molar enthalpy change *vs* molar ratio) of aptananozyme IV, with L-DOPA (i) and D-DOPA (ii) in 5 mM MES pH 5.5, 5 mM $MgCl_2$, 100 mM NaCl.

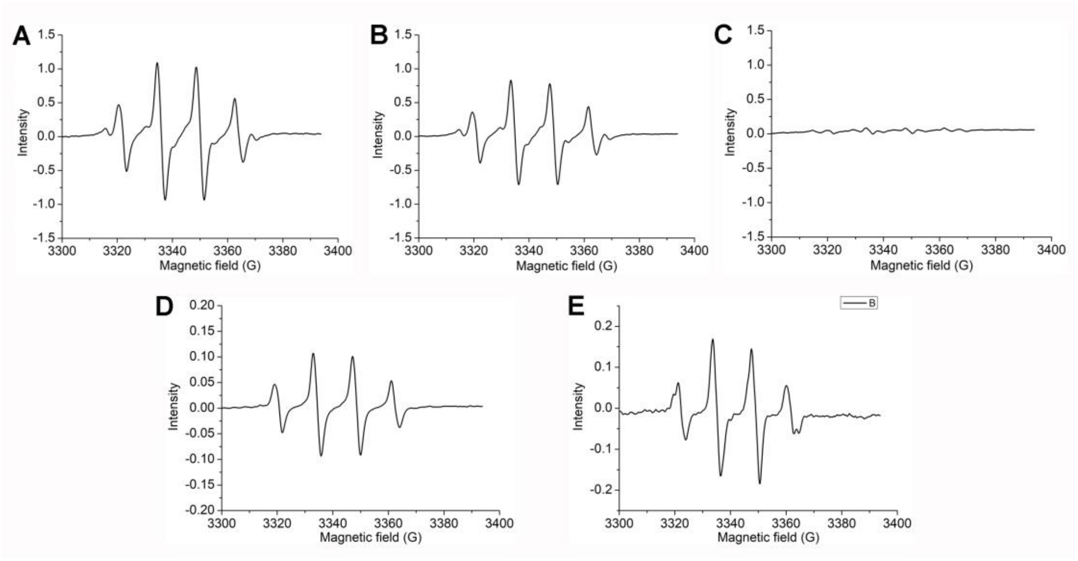

Figure S12. (A) EPR spectrum corresponding to the •OH generated by the aptananozyme II in the presence of H_2O_2. (B) EPR spectrum corresponding to the •OH generated by the aptananozyme II in the presence of glucose. (C) EPR spectrum corresponding to the •OH generated by the aptananozyme II in the presence of glucose under nitrogen. (D) EPR spectrum corresponding to the •OH generated by the aptananozyme II in the presence of H_2O_2 and dopamine. (E) EPR spectrum corresponding to the •OH generated by the aptananozyme II in the presence of glucose and dopamine.

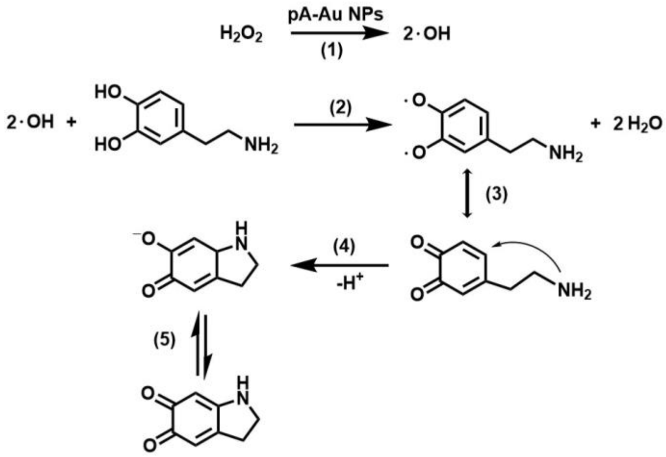

Figure S13. Schematic suggested mechanism for the oxidation of dopamine yielding the aminochrome products by DBA-functionalized pA-Au NPs, aptananozyme.

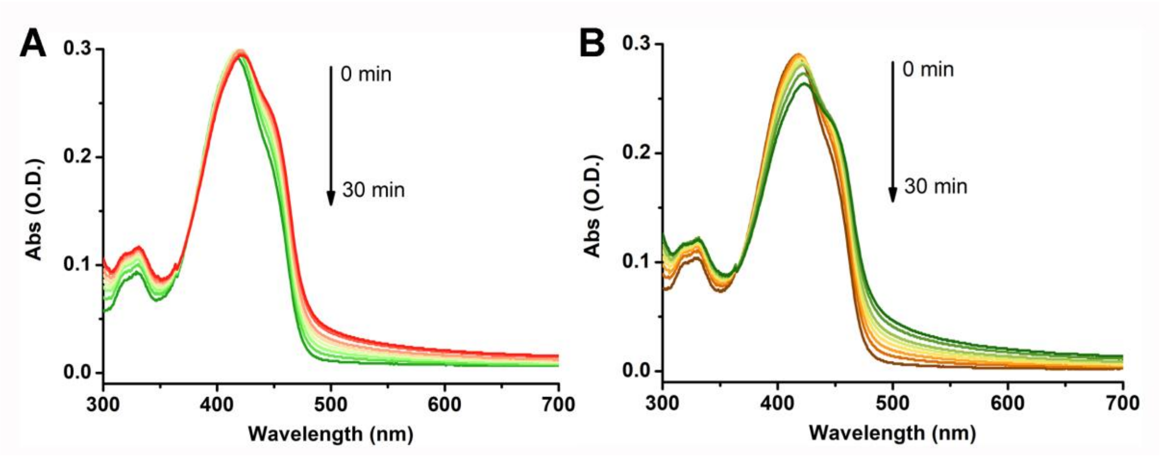

Figure S14. Time-dependent absorbance spectra upon reaction with the ROS species generated by: (A) the absence of H_2O_2. (B) the presence of H_2O_2.

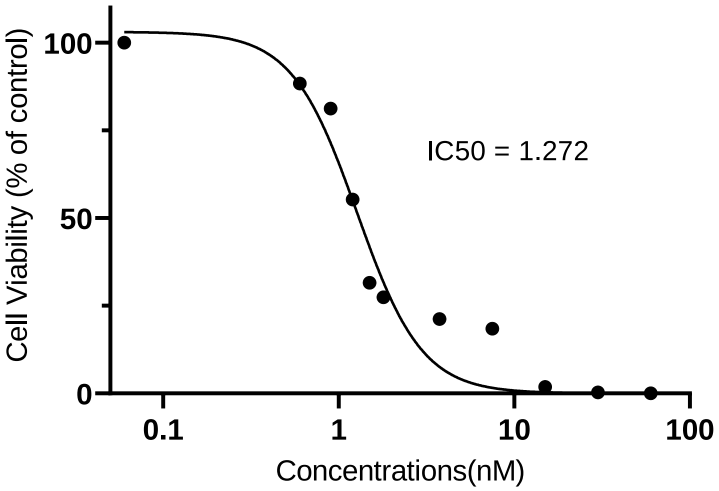

Figure S15. Viability of MDA-MB-231 cancer cells after exposure to different types of AS1411-pA-Au NPs for two days.

Table S1. The DNA strands used in the aptananozymes systems.

DNA Strands	Sequence (5′-3′)
(**1**)	CGACGCCAGTTTGAAGGTTCGTTCGCAGGTGTGGAGTGACGTCGGTAACAACAGTCAAAAAAAAA AAAAAAAAAAAGACTGTTGTTAC
(**2**)	GTAACAACAGTCAAAAAAAAAAAAAAAAAAAAGACTGTTGTTACCGACGCCAGTTTGAAGGTTC GTTCGCAGGTGTGGAGTGACGTCG
(**3**)	GTAACAACAGTCAAAAAAAAAAAAAAAAAAAAGACTGTTGTTACTGTACGACGCCAGTTTGAAG GTTCGTTCGCAGGTGTGGAGTGACGTCG
(**4**)	GTAACAACAGTCAAAAAAAAAAAAAAAAAAAAGACTGTTGTTACTGTATGTACGACGCCAGTTTG AAGGTTCGTTCGCAGGTGTGGAGTGACGTCG
(**5**)	GTAACAACAGTCAAAAAAAAAAAAAAAAAAAAGACTGTTGTTACTGTATGTATGTACGACGCCAG TTTGAAGGTTCGTTCGCAGGTGTGGAGTGACGTCG
(**6**)	GTAACAACAGTCAAAAAAAAAAAAAAAAAAAAGACTGTTGTTACGGTGGTGGTGGTTGTGGTGGT GGTGGTTT
(**2a**) (scrambled)	GTAACAACAGTCAAAAAAAAAAAAAAAAAAAAGACTGTTGTTACGACTAGCGTGTGTGATGGGAC CTTAGGCCGTCACGGGGCTTAGT
DBA	CGACGCCAGTTTGAAGGTTCGTTCGCAGGTGTGGAGTGACGTCG
pA	GTAACAACAGTCAAAAAAAAAAAAAAAAAAAAGACTGTTGTTAC
